# Heme Oxgenase-1, a Cardinal Modulator of Regulated Cell Death and Inflammation

**DOI:** 10.3390/cells10030515

**Published:** 2021-02-28

**Authors:** Stefan W. Ryter

**Affiliations:** Joan and Sanford I. Weill Department of Medicine, Weill Cornell Medical College, 525 East 68th Street, Room M-522, Box 130, New York, NY 10065, USA; str2020@med.cornell.edu or Stefan.Ryter@proterris.com

**Keywords:** apoptosis, autophagy, carbon monoxide, cell death, ferroptosis, heme oxygenase, inflammasome, inflammation, necroptosis, pyroptosis

## Abstract

Heme oxygenase catalyzes the rate-limiting step in heme degradation in order to generate biliverdin, carbon monoxide (CO), and iron. The inducible form of the enzyme, heme oxygenase-1 (HO-1), exerts a central role in cellular protection. The substrate, heme, is a potent pro-oxidant that can accelerate inflammatory injury and promote cell death. HO-1 has been implicated as a key mediator of inflammatory cell and tissue injury, as validated in preclinical models of acute lung injury and sepsis. A large body of work has also implicated HO-1 as a cytoprotective molecule against various forms of cell death, including necrosis, apoptosis and newly recognized regulated cell death (RCD) programs such as necroptosis, pyroptosis, and ferroptosis. While the antiapoptotic potential of HO-1 and its reaction product CO in apoptosis regulation has been extensively characterized, relatively fewer studies have explored the regulatory role of HO-1 in other forms of necrotic and inflammatory RCD (i.e., pyroptosis, necroptosis and ferroptosis). HO-1 may provide anti-inflammatory protection in necroptosis or pyroptosis. In contrast, in ferroptosis, HO-1 may play a pro-death role via enhancing iron release. HO-1 has also been implicated in co-regulation of autophagy, a cellular homeostatic program for catabolic recycling of proteins and organelles. While autophagy is primarily associated with cell survival, its occurrence can coincide with RCD programs. This review will summarize the roles of HO-1 and its reaction products in co-regulating RCD and autophagy programs, with its implication for both protective and detrimental tissue responses, with emphasis on how these impact HO-1 as a candidate therapeutic target in disease.

## 1. Introduction

**Figure 1:**
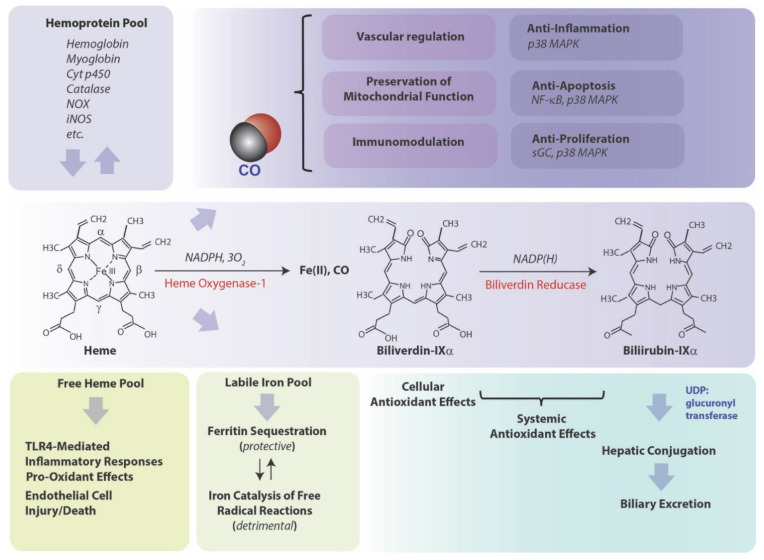
HO activity and role in cytoprotection. Heme oxygenase (HO: heme, hydrogen-donor:oxygen oxidoreductase (α-methene-oxidizing, hydroxylating), EC: 1:14:99:3) is the rate-limiting step in heme degradation. HO catalyzes the oxidative cleavage of heme at the α-methene bridge carbon, released as carbon monoxide (CO), to generate biliverdin-IXα (BV), while releasing the central heme iron chelate as ferrous iron (Fe II). Enzymatic heme degradation requires three moles of molecular oxygen (O_2_) and electrons derived from NADPH-cytochrome p450 reductase (EC: 1.6.2.4). The BV generated in the HO reaction is subsequently reduced by NAD(P)H: biliverdin reductase (BVR; EC: 1.3.1.24) to generate the lipid-soluble bile pigment bilirubin-IXα (BR). The source of heme for the HO reaction is derived from the turnover of hemoglobin and cellular hemoproteins. Free unbound heme released from hemolysis may represent a pro-oxidant hazard to vascular endothelium and may initiate pro-inflammatory reactions. BV and BR are known antioxidants, with circulating BR implicated as a mitigator of cardiovascular disease risk. Iron released from HO activity is equilibrated into ferritin storage, whereas unbound iron may propagate injury via catalysis of free radical-generating reactions. CO evolving from HO activity may modulate cellular function, via regulation of vascular function, immune system function, inflammation, apoptosis, and cellular proliferation. Abbreviations: CO: carbon monoxide; Cyt p450: cytochrome p450; iNOS: inducible nitric oxide synthase; NF-κB: nuclear factor-kappa-B; NOX: NADPH oxidase isoforms; p38 MAPK: p38 mitogen-activated protein kinase; sGC: soluble guanylate cyclase; TLR4: Toll-like receptor-4; UDP: uridine 5’-diphosphate.

Heme oxygenase (HO-1), a vital metabolic enzyme, has emerged as a central effector of the mammalian stress response [1,2]. Early studies of microsomal metabolic activities established heme oxygenase (HO) as the rate-limiting step in heme degradation [3]. HO activity catalyzes the oxidative cleavage of heme at the α-methene bridge carbon, released as carbon monoxide (CO), to generate biliverdin-IXα (BV), while releasing the central heme iron chelate as ferrous iron (Fe II) [3]. The BV generated in the HO reaction is subsequently reduced by NAD(P)H: biliverdin reductase, to generate the lipid-soluble bile pigment bilirubin-IXα (BR) (Figure 1) [4]. Cellular HO activity is provided by two major isoforms, an inducible isozyme (HO-1) and a constitutively expressed isozyme (HO-2), which have distinct biochemical properties and arise from separate genes [5,6].

The HO-1 field continues to attract worldwide research interest, from its mechanistic roles in regulating fundamental biological and metabolic processes, to its continuing status as a candidate therapeutic target in many disease states. Investigations in the mid-1980s established HO-1 as identical to a 32 kDa shock protein regulated by multiple forms of chemical and physical cellular stress, including oxidizing ultraviolet-A radiation, and heavy metals [7,8,9]. The importance of HO-1 in systemic homeostasis and iron balance was deduced from early studies using mice genetically deficient in HO-1 (*Hmox1^−/−^*). These mice were characterized by abnormal systemic iron metabolism including hepatic and renal iron deposition and anemia. Furthermore, these mice and endothelial cells derived from these mice were highly susceptible to oxidative stress [10,11]. The essential role of HO-1 in human physiology was also underscored by a unique case of HO-1 genetic deficiency in a human subject, who bore symptoms of systemic endothelial cell injury, anemia, and abnormal tissue iron accumulation [12]. Seminal studies on the macrophage inflammatory response established HO-1 as an anti-inflammatory mediator, which can limit Toll-like receptor-4 (TLR4)-dependent pro-inflammatory cytokine(s) production in activated macrophages [13]. Accumulating research since then has revealed that HO-1 can exert pleiotropic roles in mitigating inflammation, via multiple molecular mechanisms including modulation of p38 mitogen-activated protein kinase (MAPK) activity [13,14]. Furthermore, additional pioneering studies established HO-1 as an antagonist of TNF-induced endothelial cell apoptosis [15].

Cell death pathways were traditionally segregated into genetically regulated and non-regulated programs (i.e., apoptosis and necrosis, respectively) [16,17,18]. Apoptosis requires the activation of cysteine proteases (e.g., caspases) and endonucleases without loss of plasma membrane integrity, and is morphologically characterized by cytosolic shrinkage, membrane blebbing, chromatin condensation and DNA fragmentation [19]. In contrast, necrosis was defined as an accidental or catastrophic cell death characterized by loss of energy charge, cell swelling, and plasma membrane damage resulting in the leakage of cytosolic constituents into the extracellular space, and which may trigger local inflammation and damage to surrounding tissues [18].

Further emergent discoveries have elucidated novel cell death pathways, accompanied by the paradigm-shifting revelation that certain modes of cell death that share morphological features of necrosis can also be regulated by underlying genetic programs [16]. The resulting change in cell death nomenclature now groups apoptosis with distinct forms of regulated necrosis under the term regulated cell death (RCD), to exclude non-regulated necrosis now classified as accidental cell death (ACD) [16]. The newly recognized forms of RCD include ferroptosis, pyroptosis, necroptosis, and other modalities as recently reviewed [20]. An additional genetically regulated cellular program, referred to as autophagy, represents a mechanism for cellular catabolism. Autophagy was originally classified as a cell death mode due to its coincidence with RCD and may also impact the regulation of inflammation [21].

Investigation into the role of HO-1 in cell death and related pathways was initially restricted to its largely protective role apoptosis and necrosis. Emerging studies suggest that HO-1 will have a complex modulatory or regulatory role in not only apoptosis and autophagy, but also in newly uncovered forms of RCD, namely pyroptosis, necroptosis, and/or ferroptosis. This review will summarize the underlying molecular mechanisms of regulation and function that characterize HO-1 as a unique response to oxidative stress and inflammation, and as a mitigator of cell survival and cell death programs; with consideration on how these processes may ultimately impact the candidate role of HO-1 as a therapeutic target in disease.

## 2. Molecular Regulation of Heme Oxygenase-1

HO-1 expression responds to many diverse chemical and physical agents, including the substrate heme, a pro-oxidant compound, oxidants (e.g., H_2_O_2_), exposure to ultraviolet-A radiation, nitric oxide (NO), heavy metals and other thiol-reactive chemicals [2,7,8,9,14]. Further, a broad class of electrophilic plant-derived polyphenolic compounds including flavonoids and other natural antioxidants are potent inducers of HO-1 [22,23,24,25,26,27,28]. HO-1 responds to pro-oxidant states associated with enhanced reactive oxygen species (ROS) generation, as can be produced from dysfunctional mitochondria (mtROS) or activated inflammatory cells. Altered states of oxygen tension (pO_2_) above and below physiological levels can also modulate ROS production from mitochondrial metabolism. High oxygen tension (hyperoxia) increases substrate availability (O_2_) for enhanced mitochondrial ROS (mtROS) production, and/or increased NADPH oxidase enzymatic activity, represented by superoxide (O_2_^−^) production, and acts as a potent inducing signal for HO-1 [29]. In contrast, low pO_2_ (hypoxia) also favors increased ROS flux in the electron transport chain by impairing cytochrome-*c* oxidase activity, and selectively induces HO-1 in a species-specific manner, particularly in rodents [30,31]. HO-1 upregulation by these agents occurs mainly by transcriptional upregulation of the *HMOX1* gene (*Hmox1* in rodents), and results in de novo synthesis of the protein [32].

Extensive mechanistic studies have revealed that HO-1 gene regulation responds to positive regulation by nuclear factor erythroid 2-related factor-2 (Nrf2), a Cap’n’collar/basic-leucine zipper family protein that can heteromerize with small Maf proteins [33]. Nrf2 is regarded as a master regulator of the antioxidant response and regulates a series of other genes involved in detoxification. The Kelch-like ECH-associated protein (Keap1) inhibits HO-1 expression by acting as a cytoplasmic anchor for Nrf2 under basal conditions [34,35]. Keap1 enables the targeting of Nrf2 by Cullin 3-based E3 ubiquitin ligase complex, which marks Nrf2 for proteasomal degradation [36,37]. When cells are exposed to inducing stimuli, Keap1 dissociates from Nrf2, which subsequently translocates to the nucleus, where it can activate gene expression, including the *Hmox1* gene [33].

Transcription factor Bach-1 acts as a transcriptional repressor of HO-1 gene expression via competition with Nrf2 [31,38,39,40]. Heme can inhibit the DNA-binding activity of Bach-1 by direct binding, as well as promote the nuclear export of Bach-1 and inhibit the proteasomal degradation of Nrf2, hereby increasing HO-1 expression [38,39,41,42]. Both Nrf2 and Bach-1 target distinct sites located in the promoter regions of *Hmox1* genes. Comprehensive promoter analyses of the *Hmox1* gene uncovered enhancer regions located at −4 kb and −10 kb relative to the *Hmox1* transcriptional start site [43,44]. The dominant sequence element of the enhancers is the stress-responsive element (StRE), which is synonymous with the Maf response element (MARE) and antioxidant response element (ARE) [45,46]. A number of additional transcription factors have been implicated in HO-1 transcriptional regulation in a cell type-specific and inducer-specific fashion. These include AP-1 (Fos/Jun heterodimer), AP-2, heat shock factor-1 (HSF-1), hypoxia-inducible factor-1 (HIF-1), early growth-1 protein (Egr-1), nuclear factor-kappa-B (NF-κB), and cyclic AMP responsive element binding protein (CREB). The relative importance of these has been reviewed elsewhere [47,48].

In addition to regulation by transcription factor networks, emerging evidence suggests that HO-1 is post-transcriptionally regulated [49]. Several studies have implicated microRNAs (miRs) directly or indirectly in HO-1 regulation [50,51,52,53,54,55,56,57,58,59]. The miRs are small non-coding RNAs that can impact the outcome of gene expression by altering mRNA stability or translation. Previous studies have identified miR candidates that can directly or indirectly influence HO-1 expression in a context-specific fashion. For example, miR-494 was found to promote HO-1 expression under oxidative stress conditions in neurons [50]. miR-378 overexpression was shown to downregulate HO-1 coincident with promotion of cell proliferation, whereas HO-1 expression reciprocally downregulated miR-378 [51]. Other miRs identified as influencing HO-1 regulation include inhibition by miR-24/mIR-24-3p [54], miR-200c [55], miR-155 [56], and miR-377/miR-217 [57]. Recent studies also implicate miRNA-dependent regulation of HO-1 in modulation of allergic inflammation (i.e., miR-205, miR-203, and miR-483-5p) [58], and iron-dependent neuroinflammation (miR-183-5p) [59].

Importantly, miRs can also indirectly regulate HO-1 via regulating the expression and/or stability of its upstream regulatory molecules, such as Nrf2 [55,60,61,62,63,64], or its cytoplasmic anchor molecule Keap1 [65,66]. For example, miR-101 promoted Nrf2 expression via inhibition of its ubiquitination [62], whereas miR-141-3p and miR200a were found to target Keap1, resulting in indirect activation of Nrf2 and HO-1 [65,66]. Several miRs (e.g., miR-155, mIR-196, let-7, miR-98-5p) can influence HO-1 expression through the downregulation of the transcriptional repressor Bach-1 [67,68,69,70]. HO-1 has also been implicated as an upstream functional influencer of miR networks, which in turn implicate downstream miR-dependent effects as possibly mediating the functional effects of HO-1 in various biological processes, including differentiation, angiogenesis, cell proliferation, inflammation and tumorigenesis [71,72]. For example, expression of HO-1 in myoblasts led to inhibition of specific (myo)miRs (e.g., miR-1, miR-133a/b, and miR-206) associated with inhibition of myoblast differentiation [73]. An effect of the non-canonical nuclear HO-1 (NHO-1) on blood–spinal cord barrier integrity was attributed to regulation of miR-181c-5p [74]. HO-1 overexpression has also been associated with promotion of rhabdosarcoma tumors via a mechanism involving HDAC4 nuclear localization and downregulation of miR-206. Conversely, inhibition of HO-1 activity or of HDAC4 activated a myogenic program via upregulation of miR-206 [75].

Taken together, these observations highlight an increasing complexity of HO-1 regulation and function in which miR-dependent regulation has now been implicated.

Accumulating genetic epidemiology studies have suggested that non-coding polymorphisms can occur in the *HMOX1* gene and impact gene regulation in carriers. Microsatellite (GT)_n_ dinucleotide length polymorphism were found to occur in the promoter region of the human *HMOX1* gene, which can inhibit transcriptional regulation and HO-1 expression in carriers of the long (L) allele [ie., (GT)n ≥ 30] [76]. Several studies have described associations with the L allele and susceptibility or severity to cardiovascular diseases (CVD), including coronary artery disease (CAD) and atherosclerosis [77,78,79]. Subjects homozygous for (GT)n ≥ 32 had greater CVD risk, enhanced atherosclerosis progression, and a trend toward increased oxidative stress biomarkers [77]. Recent studies indicate that CAD patients with reduced ejection fraction had longer *HMOX1* promoter (GT)_n_ repeats than those with mid-range ejection fraction. The presence of L-allele was a predictor for diagnosis of low ejection fraction in CAD [78] and susceptibility to CAD among diabetics [78]. Additional studies describe associations of (GT)n ≥ 30 alleles with critical conditions such as acute respiratory distress syndrome (ARDS) [80], and sepsis-induced acute kidney injury [81], preeclampsia [82], and risk of Type 2 diabetes [83].

In studies of chronic lung disease, L alleles of the (GT)n repeat (variable lengths but typically ≥30) were correlated with COPD susceptibility [84], emphysema [76], and COPD severity (Chinese cohort) [85,86]; with responsiveness to antioxidant therapy [87], lung function decline in COPD [88], and lung function decline in heavy smokers [89]. However, independent validation studies either failed to find association of *HMOX1* polymorphisms with lung function decline in smokers [90] or reported differential association of (GT)n = 30, but not (GT)n = 31 [91]. Lymphoblastoid cells for the L allele were more susceptible to oxidant-mediated apoptosis in culture, than cells isolated from carriers of short alleles [92]. Taken together, these investigations suggest that genetic variants in *HMOX1* gene promoter regions that inhibit gene expression may arise in subpopulations and may be linked to increased susceptibility to oxidative stress and related diseases. Additional studies will be required to prove these associations in a disease-specific manner.

## 3. HO-1-Mediated Cytoprotection, a Coordinated Protective Stratagem Based on Heme Removal and Heme Breakdown Product Generation

Since its discovery in 1968 [3], and its identification as a stress protein in 1988 [7], the mechanism(s) by which HO-1 can confer protection in cells and tissues, in the context of its induction by stress stimuli, remain partially understood. As the degradation of heme is the primary enzymatic function of HO-1, it stands as a valid hypothesis that its function in hemoprotein turnover and heme removal represents a cardinal mechanism underlying cytoprotection [93,94,95,96]. Indeed, heme, which has a central iron atom, has been implicated as a pro-oxidant and catalyst of free radical-generating reactions [93,97], a cytotoxic molecule with respect to vascular endothelial cells [98,99], and a pro-pathogenic mediator of diseases such as sepsis, and malaria [100,101]. Thus, the removal of heme by HO-1 may serve a context-specific protective and antioxidant function, via precluding heme from aggravating injurious or pathological processes [93,94,95]. By degrading heme, HO releases heme iron [1], which itself can present harmful sequelae unless detoxified, including potential catalysis of Fenton chemistry, and production of ROS and lipid peroxides [102,103]. HO-derived iron has been associated with the regulation of de novo ferritin synthesis, which in turn was associated in adaptive cytoprotection against pro-oxidant stimuli such as UVA radiation [104,105]. Ferritin is a complex multimeric molecule consisting of H and L chains which sequesters intracellular redox active iron in a crystalline core [106]. Ferritin has been characterized as a cytoprotective molecule in the vascular endothelium [107,108,109]. HO activity also releases BV which in turn is reduced to BR [3]. Both BV and BV have been shown to possess antioxidant properties in serum and bile and can attenuate free radical-generating reactions [110,111,112,113]. Much research has focused on the biological properties of CO, which originates from the α-methene bridge of heme during HO-mediated heme catalysis [3], and has emerged as an endogenous gaseous signaling mediator. CO derived from HO-1 activity was implicated in anti-inflammatory effects in macrophages based on the modulation of p38 MAPK activity. Evidence has accumulated that low concentration CO, when applied exogenously, can confer cyto- and tissue protection in inflammatory disease models in effect by influencing inflammation, apoptosis, and cell proliferation programs [2,14,114,115]. Under conditions where HO-1 is associated with cytoprotection, the pleiotropic effects of HO-1 may represent a complex cooperation of the generation and distribution of bioactive catabolic products and their downstream effects [2,14,116]. To achieve these cytoprotective effects, HO-1 expression must be tightly regulated. In contrast, detrimental functional roles of HO activity have been ascribed to iron overload effects [117,118], and may be relevant in neurodegenerative diseases [119].

## 4. Non-Canonical Roles for HO-1-Mediated Protection

**Figure 2:**
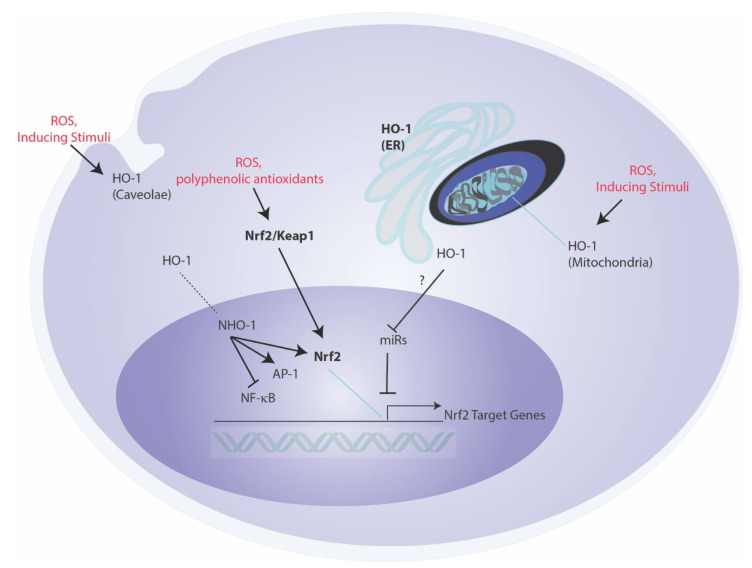
Subcellular localization and non-canonical roles of HO-1. Endoplasmic reticulum (microsomal)-associated HO-1 induction by chemical and physical stress represents the classical form of HO-1 regulation and is implicated in cellular homeostasis and cytoprotection. Additional compartment-specific subcellular localization of HO-1 has been described including stress activated translocation of HO-1 to the mitochondria. The HO-1 likely functions in regulating heme bioavailability in this compartment/and or localized CO production. HO-1 has also been shown to localize to plasma membrane caveolae, where it forms an inhibitory complex with caveolin-1. HO-1 may also migrate to the nucleus in a COOH-terminal truncated nuclear form (NHO-1) that is devoid of enzyme activity. This NHO-1 has been implicated in transcription factor regulation, including NF-κB, AP-1, and Nrf2, the latter which regulates the antioxidant response, including HO-1 gene expression. Abbreviations: AP-1: activator protein-1; ER: endoplasmic reticulum; HO-1: heme oxygenae-1; miR: microRNA, NF-κB: nuclear factor-kappa-B; NHO-1: nuclear HO-1, Nrf2: NF-E2-related factor-2; ROS: reactive oxygen species.

An emerging hypothesis suggests that the biological and cellular functions of HO-1 may in part relate to specific subcellular compartmentalization, and/or may transcend the catalytic breakdown of heme, with certain effector functions that are independent of its enzymatic reaction products (Figure 2) [120,121]. While limited studies support this notion, this may include possible intermolecular interactions between other cellular proteins, that influence the function of other signaling proteins in an activity-independent fashion. One of these proposed interactions is that of HO-1 with CD91 [121]. An intermolecular interaction of HO-1 with the pro-apoptotic molecule Bax was described in a proposed antiapoptotic mechanism in the context of endothelial cell injury [122]. HO-1 is also reported to translocate to the nucleus under stress conditions, where it may influence nuclear function [123]. The nuclear form of HO-1 (NHO-1) is reported to exist in a truncated form (28 kDa), and to be devoid of heme-degrading activity [124]. NHO-1 has been described as a regulator of nuclear transcription factor activities, as exemplified by NF-κB p65, AP-1, and Nrf2-dependent activities [121]. The bimolecular interaction of NHO-1 with Nrf2 was shown to prevent GSK3β-mediated phosphorylation of Nrf2 and proteolytic degradation, thereby stabilizing Nrf2 [124]. The NHO-1-mediated stabilization of Nrf2 was shown to promote transcriptional regulation of several Nrf2 target genes, including NQO1 and G6PDH [124]. Recent studies indicated that artificial overexpression of NHO-1 (COOH-terminal truncated form) can confer protection in a model of blood–spinal cord barrier integrity, by the downstream modulation of miR-181c-5p and SOX5-mediated upregulation of tight junction protein expression [74].

In the nucleus, a relationship between HO-1 and regulation of G-quadruplexes has been proposed. G-quadruplexes (G4) refer to stacked nucleic acid secondary structure in guanine-rich regions of DNA, that have a high affinity for heme-binding and are stabilized by heme. Hematopoietic stem cells (HSCs) derived from *Hmox1^−/−^* mice displayed increased expression of G4-unwinding helicases (e.g., *Brip1* and *Pif1*) and reduced G4 content. In contrast, induced pluripotent stem cells (iPSCs) derived from *Hmox1^−/−^* mice also displayed increased helicase expression, but with more G4 content, which was increased by exogenous heme application [125]. Selective expression of the nuclear isoform NHO-1 was shown to result in reduction in G4 content in the nucleus [125]. Additionally, EPhenDC3, a non-heme G4 ligand, was found to displace quadruplex-bound heme in vitro, and to dramatically induce HO-1 gene expression in human cells [126]. Taken together, these experiments suggest a relationship between HO-1 and degradation of heme released from G4 complexes. Further experimentation will elucidate these relationships.

In addition to nuclear localization, evidence has accumulated for localization of HO-1 in other subcellular compartments. Specifically, a mitochondrial localization of functionally active HO-1 has been reported under stress conditions, and which may regulate heme bioavailability for mitochondrial cytochromes [127]. Localization of HO-1 to plasma membrane caveolae, and intermolecular interactions with the caveolae resident scaffolding protein caveolin-1 were also described [128,129]. The caveolin-1 interaction with HO-1 was shown to inhibit HO activity, thus potentially serving as a brake on HO-1 function in this compartment [128,129]. It is plausible that HO-1 serves as a localized source of CO production in either mitochondrial or caveolae compartments for discrete signaling processes, though the functional significance of these localization events remain incompletely understood [127,128]. Finally, non-canonical roles of HO-1 have been suggested to include possible roles of a circulating cell free form of HO-1 in the extracellular space [120]. HO-1 has been detected in both serum and cerebrospinal fluids and to vary with disease, thought the functional significance of HO-1 in extracellular fluids remains unclear [120].

## 5. HO-1 as a Regulator of Inflammation

HO-1 expression, in particular that of macrophages and inflammatory cells, can potentially modulate the acute inflammatory response, via several proposed mechanisms, including modulation of TLRs dependent regulation of cytokine gene expression, as well as other innate immune mechanisms such as regulation of inflammasome-dependent cytokine maturation, macrophage polarization, and resolution of inflammation [2,13,14,130]. Anti-inflammatory effects of HO-1 were originally demonstrated using in vitro and in vivo models of inflammation and acute lung injury (ALI) [13,131]. Adenoviral-directed HO-1 gene expression inhibited bacterial lipopolysaccharide (LPS)-induced production of pro-inflammatory cytokines, including tumor necrosis factor-α (TNF-α), interleukin (IL)-1β, IL-6, and macrophage inflammatory protein-1β (MIP-1β) in cultured macrophages, as well as increased the production of the anti-inflammatory cytokine IL-10 during LPS challenge [13]. Enhanced gene expression of HO-1 ameliorated LPS-induced lung injury in mice via increased IL-10 production [13,132]. Enhanced gene expression of HO-1 in lungs by intratracheal adenoviral-mediated gene transfer also limited murine ALI in response to influenza virus infection [133]. Conversely, HO-1 was also shown to be upregulated by IL-10 expression, suggesting that the anti-inflammatory effect of this cytokine may depend on reciprocal HO-1 activation [134]. Pharmacological application of individual HO reaction products BV, or of CO (250 ppm) also inhibited pro-inflammatory cytokine production, upregulated IL-10 levels, and reduced ALI in LPS-treated rodents [13,135]. HO-1 genetically deficient mice (*Hmox1*^−/−^) were found susceptible to the lethal effects of cecal-ligation and puncture (CLP)-induced polymicrobial sepsis, compared with wild-type mice [100]. The *Hmox1*^−/−^ mice displayed elevated levels of free circulating heme and reduced levels of the heme-binding protein hemopexin, rendering them more susceptible sepsis-induced mortality [100]. The enhanced inflammation associated with sepsis was also attributed to increased levels of high-mobility group box-1 protein (HMGB1), which was augmented by *Hmox1* deficiency, and ablated by pharmacological upregulation of HO-1 or application of CO [136,137].

A role of HO-1 (and HO-derived CO) in bacterial clearance during sepsis has also been proposed. Targeted overexpression of HO-1 to smooth muscle cells and myofibroblasts, and bowel was shown to protect against sepsis-induced mortality associated with *Enterococcus faecalis* infection. HO-1-mediated protection in sepsis models was associated with enhanced bacterial clearance via increased phagocytosis and the endogenous antimicrobial response [138]. Application of CO (250 ppm, pre- or post-treatment) also protected mice against polymicrobial sepsis, via stimulating the autophagy pathway and promoting bacterial clearance by macrophages. The candidate mechanisms by which exogenous CO modulates the regulation of inflammation and autophagy have been reviewed elsewhere [2,14].

### 5.1. Inflammasome Regulation

HO-1 and its reaction product CO have been implicated in modulation of innate immune responses. Inflammasomes are specialized macromolecular protein complexes that reside in immune cells and which regulate the proteolytic cleavage of caspase-1. In turn, caspase-1 is responsible for the maturation and secretion of pro-inflammatory cytokines including interleukin-1β (IL-1β) and IL-18. The NOD-, leucine-rich region- and pyrin domain-containing-3 (NLRP3)-dependent inflammasome has been implicated in the pathogenesis of several acute or chronic inflammatory diseases [139]. 

Heme pre-conditioning to induce HO-1 can reduce IL-1β maturation and downregulate inflammasome activation in a model of sepsis-associated lung injury [140]. Furthermore, induction of HO-1 by heme conditioning was associated with protection from acute liver injury induced by D-galactosamine and LPS, and with downregulation of the associated NLRP3 inflammasome-dependent activation of caspase-1 [141]. CO, when applied exogenously (250 ppm) was found to downregulate NLRP3 inflammasome activation in bone marrow-derived macrophages (BMDM) stimulated with LPS and ATP. Contrasting studies found that CO can upregulate NLRP3 activation in the presence of live bacteria via mobilization of ATP from bacteria [142,143].

### 5.2. Macrophage Polarization

Macrophages can be classed in subpopulations (M1, M2) whose distribution change during exposure to environmental or inflammatory stimuli. M1 macrophages (classically activated macrophages) are responsive to proinflammatory cytokines such as IFN-γ, and TNF-α, produce pro-inflammatory cytokines, eliminate intracellular pathogens via phagocytosis, and promote a local Th1 environment. M2 macrophages (alternatively activated macrophages) are responsive to IL-4 and IL-13, and regulate Th2 immune responses, incluing anti-inflammatory cytokine (IL-10) production. Delayed accumulation of M2 macrophages may contribute to tissue fibrosis or repair [144].

HO-1 has been implicated as a modulator of immune responses via the promotion of macrophage M2 macrophage polarization. HO-1 was found to be highly expressed in several M2 macrophages subsets, while HO-1 elevation by various inducing stimuli can drive the phenotypic shift to M2 macrophages [145]. For example, HO-1 expression was higher in M2 macrophages induced by M-CSF, relative to M1 macrophages induced by GM-CSF. HO-1 expression in M-CSF-induced M2 macrophages responded to IL-4 stimulation. Further, HO-1 inducing stimuli such as cobalt protoporphyrin-IX, augmented LPS-stimulated production of the pro-inflammatory cytokine IL-10 from M2 macrophages. In metastatic melanoma, HO-1 expression was high in CD163(+) tumor-associated macrophages, which are primarily M2 polarized [146].

In recent genetic validation studies, BMDMs isolated from myeloid-specific HO-1–knockout (mHO-1–KO), treated with M1-inducing (i.e., LPS) or M2-inducing (i.e., IL-4) ligands, exhibited increased gene expression of M1 markers (i.e., CXCL10, IL-1β and MCP1) and decreased expression of M2 markers (i.e., Arg1 and CD163) [147]. These experiments support the hypothesis that HO-1 promotes the M2 phenotype. In a murine model of hepatic IRI, mHO-1–KO mice displayed similar decrease in M2 phenotype, in association with increased susceptibility to IRI. Opposing findings were observed in HO-1 overexpressing transgenic (mHO-1–Tg) mice, with promotion of an M2 phenotype and protection in hepatic IRI. Human liver transplant biopsies revealed increased HO-1 levels in association with reduced M1 markers [147].

### 5.3. Inflammation Resolution

HO-1 and its reaction product CO have also been implicated as regulators of the resolution phase of inflammation [148]. In mice, inhaled CO (250 ppm) inhibited peritoneal neutrophil infiltration and shortened resolution interval after zymosan challenge. CO reduced leukotriene B4 (LTB4) and increased the production of specialized pro-resolving mediators (SPMs) including resolvin (RvD1) and maresin-1. In human macrophages, exposure to SPM increased HO-1 expression. CO also enhanced HO-1 expression and accumulation of RvD1 and RvD5, and these events were reversed by inhibition of 15-lipoxygenase type-1 (15-LOX-1). CO increased phagocytosis by human macrophages, which was further enhanced by SPM, and antagonized by 15-LOX-1 inhibition. SPM stimulated phagocytosis was reduced by inhibition of HO-1. In a murine peritonitis model, CO inhalation increased macrophages efferocytosis and enhanced PMN apoptosis. This study suggested pro-resolving mechanisms for HO-1 and CO, in SPM-initiated resolution of inflammation [148].

### 5.4. Role of Heme Oxygenase-2 (HO-2) in Inflammation

Whereas HO-1-mediated regulation of inflammation is typically associated with induction by various stimuli and cellular factors, HO-2, which is typically not regulated by HO-1-inducing stimuli, has been implicated in the regulation of inflammation.

Mice genetically deleted for HO-2 (*Hmox2^−/−^*) displayed a phenotype of exaggerated inflammatory response in zymosan-induced peritonitis [149]. HO-2 deletion was associated with enhanced peritoneal and corneal inflammation, impaired resolution of inflammation, and reduced HO-1 expression in peritoneal leukocytes [149].

Genetic deletion of *Hmox2* in mouse aortic endothelial cells (mAEC) resulted in elevated expression of vascular endothelial growth factor receptor 1 (VEGFR1) and increased angiogenic response relative to wild-type cells. Furthermore, *Hmox2^−/−^* mAEC displayed increased pro-inflammatory responses including NF-κB activation and pro-inflammatory cytokines (i.e., IL-1α and IL-6) production. The authors concluded that HO-2 deletion promotes a pro-inflammatory, pro-oxidative, and pro-angiogenic phenotype [150].

In cerebral microvascular endothelial cells (CMVEC), HO-2, which is highly expressed in this cell type, was observed to regulate the expression of pro-inflammatory cytokines. Overexpression of HO-2 inhibited TLR4/myD88-dependent proinflammatory cytokine expression (i.e., TNF-α and IL-6) in CMVEC [151]. CMVEC isolated from Hmox2^−/−^ mice were also sensitized to apoptosis in response to serum deprivation and TNF-α relative to wild-type CMVEC [152]. In a model of injury to corneal epithelium, *Hmox2^−/−^* mice displayed delayed corneal wound closure associated with neutrophil influx [153]. Furthermore, *Hmox2^−/−^* mice displayed delayed wound closure and reduced vessel density and collagen deposition in a mouse model of incisional wound healing [154]. Skin grafts taken from HO-2 overexpressing mice displayed reduced macrophage inflammatory responses and improved graft survival in a skin transplantation model [155]. Taken together, these data suggest that HO-1 participates basally in the regulation of inflammatory responses.

## 6. Relationship of Autophagy and HO-1: Two Tandem Cytoprotective Mechanisms?

### 6.1. Autophagy Process and Regulation

Autophagy is a genetically regulated cellular program that functions in the lysosome-dependent degradation of cellular organelles and denatured or long-lived proteins [21,156,157,158,159,160]. During this process, cytoplasmic substrates are compartmentalized in double-membrane bound vesicles called autophagosomes. Cargo-laden mature autophagosomes fuse to lysosomes, forming single-membraned autolysosomes, where the delivered cargo is degraded by lysosomal proteases and other enzymes [21,156,157,158,159]. Degradation of macromolecular substrates promotes recapture of precursor molecules (i.e., amino acids, lipids, and nucleotides) for use in anabolic pathways, as a form of metabolic recycling. Autophagy is genetically regulated by a distinct series of autophagy-related genes (ATGs) whose protein products form a complex regulatory network [160,161]. Among these, Beclin-1 (Atg6) is a master regulator of autophagy, while LC3 (Atg8) and related analogs are integral to autophagosome formation [160,161].

Autophagy is generally recognized as a protective mechanism in response to nutrient deprivation. However, activation of regulated cell death pathways may be dependent on or cross regulate the autophagy program. The term “autophagic cell death” is no longer used and has been replaced with the concept that autophagy can occur contextually in dying cells [21,162,163].

Autophagy can be directed toward specific substrates in processes known as selective autophagy [160,164,165]. The ubiquitination of subcellular targets represents a universal signal for demarcation of selective autophagy substrates [165,166]. The targeting of autophagy substrates to the autophagosome is assisted by cargo adaptor proteins, including p62*^SQSTM1^* (p62) and other proteins, that can associate with ubiquitinated substrates and with ATG8 homologs at the autophagosome membrane via the LC3-interacting region (LIR) [166]. Selective autophagy programs have been identified for many types of cellular constituents and named after their specific cargo. For example, lipophagy refers to the selective degradation of lipids, xenophagy refers to pathogen-selective autophagy, while “mitophagy” denotes the selective autophagy-dependent turnover of dysfunctional mitochondria [167]. The regulation of mitophagy involves a canonical pathway dependent on the activation of the transmembrane Ser/Thr kinase PINK1 (phosphatase and tensin homolog deleted in chromosome 10 (PTEN)-induced putative kinase-1). During mitophagy activation, PINK1 is stabilized on damaged or depolarized mitochondria. Pink1 phosphorylates ubiquitin, which activates the E3: ubiquitin ligase Parkin (PARK2), which then acts to ubiquitinate targets on the mitochondrial outer membrane, as a marker for organelle degradation. Recent advances suggest that PINK1 recruits the mitophagy adaptors NDP52 and optineurin, which initiate mitophagy via ULK1 and other proteins [168].

Autophagy is tightly regulated by metabolic signals sensitive to energy levels, reducing equivalents, and nutrient status, including growth factors, glucose, and amino acid levels [157]. Growth factors negatively regulate autophagy through the mechanistic target of rapamycin (mTOR) pathway. The autophagy pathway is positively regulated by energy depletion through activation of the 5’-AMP activated protein kinase (AMPK), which senses cellular AMP levels [157]. Depletion of cellular reducing equivalents regulates autophagy via activation of the NAD^+^-dependent class III histone deactylase sirtuin 1 (SIRT1). SIRT1 can bind and catalyze the deacetylation of key autophagy regulator proteins [169].

### 6.2. Autophagy and HO-1 Cross Talk

**Figure 3:**
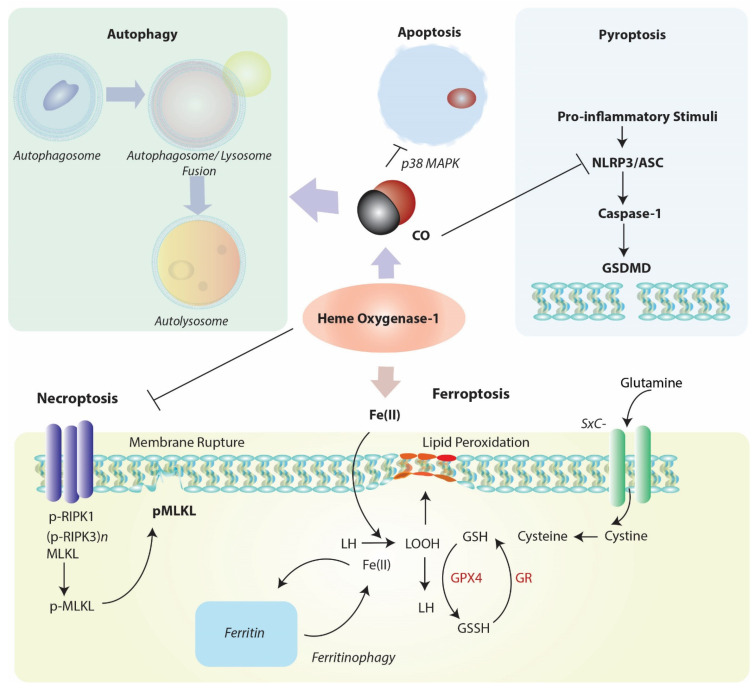
Positioning of HO-1 in relation to cellular homeostatic, apoptotic, and regulated cell death (RCD) programs. Heme oxygenase-1 and/or its reaction product CO can modulate the cellular autophagy program, which degrades cytosolic proteins and organelles, and is itself implicated in cellular survival/protection. HO-1 via its reaction product CO has been identified as a cellular antiapoptotic mediator via regulation of p38 MAPK and other factors. Induction of HO-1 via either heme clearance and/or CO production may act as a mediator of inflammatory RCD programs. Among these, HO-1-derived CO may inhibit NLRP3-ASC-mediated caspase-1 activation, which in term regulate gasdermin-D (GSDMD)-initiated pyroptosis. HO-1 dependent heme clearance may play a protective role in regulating RIPK3/MLKL-dependent necroptosis. Finally, HO-1-derived iron may promote lipid peroxidation, leading to ferroptotic cell death which implicates HO-1 as a pro-death regulator in the context of iron overproduction. This pathway is counter-regulated by ferritin, which sequesters iron, and promoted by autophagy-dependent degradation of ferritin (ferritinophagy). Abbreviations: ASC: apoptosis-associated speck-like protein containing caspase-activation and recruitment doman [CARD]; GSDMD: gasdermin-D; GSH: glutathione (reduced form); GSSG: glutathione disulfide (oxidized form); GPX4: glutathione peroxidase-4; GR: glutathione reductase; LH: lipid (reduced form); LOOH: lipid hydroperoxide; MLKL: mixed-lineage kinase domain-like pseudokinase (p-, denotes phospho- form); NLRP3: nucleotide-binding domain, leucine-rich-containing family, pyrin domain-containing-3; RIPK1: receptor-interacting serine/threonine-protein kinase 1; RIPK3: receptor-interacting serine/threonine-protein kinase-3 (p-, denotes phospho- form); SXc-: cystine/glutamate transporter, System Xc-.

Emerging evidence suggests that HO-1 is co-regulated with cellular autophagy (Figure 3), as both events as considered part of a global stress response. The nature of this relationship, which is supported by evidence of cross talk, remains incompletely understood and is explored in the following sections.

Cytotoxic agents that can promote oxidative stress and mitochondrial dysfunction may represent a common overlapping stimulus for both autophagy activation and HO-1 induction. Genetic studies have revealed that HO-1 can confer protection in part by regulating mitochondrial homeostasis via enhancing mitophagy and mitochondrial quality control. Cardiomyocyte-specific *Hmox1* deleted mice (cm-*Hmox1*^−/−^) were highly susceptible to cardiac injury when exposed to hyperoxia challenge [170]. The cm-*Hmox1*^−/−^ also displayed abnormal mitochondria. In hearts from these mice, both the PGC-1α and nuclear respiratory factor-1 (NRF1) signaling axis was inhibited. Further, mitochondrial biogenesis, and Pink1/Parkin-dependent mitophagy were functionally impaired in these mice [170]. In a model of epithelial cell injury in response to cigarette smoke (CS) exposure, activation of the autophagy program correlated with epithelial cell apoptosis [171]. In epithelial cells, viral-mediated overexpression of HO-1 reduced the expression and activation of the autophagy marker microtubule-associated protein-1 light chain 3B (LC3B), consistent with antiapoptotic cytoprotection and reduced activation of the extrinsic apoptosis pathway. siRNA-dependent HO-1 knockdown enhanced markers of autophagy and reduced cell survival in this model [171]. These results unexpectedly demonstrated that autophagy was co-regulated with apoptosis and promoted rather than protected against cell death in response to CS exposure.

HO-1 was shown to upregulate autophagy in hepatocytes, leading to protection against hepatocyte cell death and hepatic injury from infection during sepsis in mice [172]. HO-1 and autophagy were co-regulated in the liver in response to sepsis and inhibited hepatocyte cell death. Pharmacological inhibition of HO-1 activity or knockdown of HO-1 prevented the induction of autophagy and associated signaling in this model and resulted in increased hepatocellular injury, apoptosis, and hepatocyte death [172]. Recent studies demonstrate that viral-mediated HO-1 overexpression can directly induce autophagy in the liver and isolated hepatocytes, and can protect against hepatotoxin exposure [173]. Additional reports associate HO-1 dependent activation of autophagy with protection in hepatic ischemic preconditioning, I/R injury, and transplant-associated I/R injury [174,175,176]. HO-1 dependent autophagic signaling exerted anti-inflammatory effects in LPS-activated macrophages where HO-1 and autophagy cooperated to inhibit pro-inflammatory cytokine production [177]. Interestingly, the p38 MAPK inhibitor SB202190 activated autophagy and induced HO-1 in endothelial cells, in a manner that could be reversed by the autophagy inhibitor Bafilomycin A1 [178].

In addition to these observations, it is known that direct application of CO, a by-product of HO activity, can induce markers of autophagy in cultured human epithelial cells. CO exposure (250 ppm) increased the expression and lipidation of the autophagy protein LC3B in mouse lung and in cultured human alveolar or bronchial epithelial cells. Moreover, CO exposure increased autophagosome formation in pulmonary epithelial cells via upregulation of mtROS formation [179]. CO exposure also conferred protection against CLP in the mouse model associated with induced autophagy and phagocytosis, a reduction in inflammation, and enhanced bacterial clearance from organs and blood. These pro-survival effects of CO in CLP required Beclin-1-dependent autophagy [180]. Interestingly, inhibition of miR-34a provided protection in CLP sepsis via inhibition of the inflammatory response, which involved coordinated upregulation of autophagy and HO-1 [181]. These intriguing associations prompt further study into the relationship between autophagy, HO-1 directed heme metabolism, and generation of HO-1-derived CO.

## 7. HO-1 as a Modulator of Regulated Cell Death (RCD) Programs

### 7.1. HO-1 as a Modulator of Apoptosis

Apoptosis, also formally known as Type 1 programmed cell death (PCD), is the classical genetically regulated cell death (RCD) program initially discovered in *C. elegans*. Apoptosis provides essential homeostatic functions in regulating growth and development of organs, and in tissue responses to injurious stimuli, such as exposure to xenobiotics or adverse environmental conditions [17]. Disruption of apoptosis can promote tumorigenesis or autoimmune disease, whereas excessive apoptosis may cause organ failure. In fibroblasts, an antiapoptotic effect was also observed with HO-1 overexpression [182]. Application of the HO-1 reaction product CO was shown to inhibit tumor necrosis factor-α (TNFα)-initiated apoptosis in mouse fibroblasts [183], and endothelial cells [15]. The antiapoptotic effect of CO in endothelial cells required the p38 MAPK pathway [15], and downstream activation of NF-κB [183]. In cultured vascular smooth muscle cells, CO inhibited cytokine (TNFα, IL1-β, INFγ)-induced apoptosis, dependent on activation of soluble guanylate cyclase (sGC) [184,185]. Further studies revealed similar antiapoptotic effects of CO on endothelial cell apoptosis in hyperoxia [122], and in anoxia/reoxygenation models [186]. The mechanisms underlying the antiapoptotic effects of CO have been reviewed extensively elsewhere.

Human bronchial epithelial cells (Beas-2B) subjected to cigarette smoke extract (CSE) responded with a time- and dose-dependent upregulation of HO-1. At low concentrations of CSE, expression of HO-1 was shown to inhibit the activation of the extrinsic apoptotic pathway in Beas-2B cells by inhibiting the formation of the Fas-associated death-inducing signaling complex and activation of downstream caspases -8,-9,-3 [171]. Interestingly, HO-1 expression also inhibited the expression of autophagy proteins, LC3B and Beclin 1. These autophagic proteins were critical mediators of the initiation of extrinsic apoptosis in these cells in response to CSE exposure. Knockdown of either LC3B or Beclin 1 inhibited CSE-induced activation of DISC formation and caspase-8 activation in Beas-2B cells. These experiments suggest that the complex interplay of signaling molecules affected by HO-1 include not only regulators of apoptosis pathways, but also of autophagy pathways. In the case of CSE-induced cell death, enhanced autophagy correlated with increased cell death, therefore the homeostatic function of HO-1 was consistent with the downregulation of both pathways.

### 7.2. Relationship between HO-1 and Pyroptosis

Pyroptosis refers to a form of inflammation-associated programmed cell death that occurs in inflammatory cells such as macrophages and requires the activation of caspase-1. This lytic form of cell death occurs during host infection under conditions of inflammasome activation [187,188]. During pyroptosis, cells rupture to release their contents, which include excess pro-inflammatory cytokines which further propagate inflammation. Cell lysis is triggered by caspase-1-dependent activation of gasdermin-D (GSDMD) which binds plasma membrane lipids (phosphatidylinositol 4-phosphate and phosphatidylinositol 4,5-bisphosphate) and forms transmembrane pores.

During acute inflammation, pyroptosis acts as a host defense mechanism to limit infection through the elimination of infected macrophages, and to trigger host defense [189]. Excessive activation of pyroptosis can lead to tissue injury and pathogenic processes in the context of chronic inflammation [188].

Activation of the Nrf2/HO-1 pathway has been implicated as an inhibitor of pyroptosis in various model studies. The mechanisms by which HO-1 can mediate pyroptosis remain unclear but likely act at the level of inflammasome regulation. In model studies, exogenous CO application was found to inhibit caspase-1 activation and pro-inflammatory cytokines production in cultured macrophages in an in vitro model of NLRP3 inflammasome activation. Upregulation of HO-1 was associated with protection from LPS-induced acute kidney injury and caspase-1 dependent pyroptosis, in a mechanism involving PINK1 upregulation and preservation of mitochondrial function [189]. In a murine model of renal I/R injury, pyroptosis was induced in injured tissue, in association with downregulation of HO-1 [190]. Inhibition of protein arginine methylation transferase 5 (PRMT5), induced the Nrf2/HO-1 axis in conjunction with reduced tissue and cellular oxidative stress, and reduced kidney pyroptosis markers [190]. Similarly, lung I/R injury in mice was associated with activation of pulmonary macrophage pyroptosis. The application of rHBGB1 as a preconditioning agent remediated lung injury and reduced markers of pyroptosis, in a manner dependent on activation of the Nrf2/HO-1 axis [191]. In the hemorrhagic shock and resuscitation (HSR) model, application of CO also inhibited pyroptosis [192]. Taken together, these studies suggest that HO-1/CO can inhibit inflammatory cell death in injury models, but more studies are needed to determine the mechanisms by which HO-1 or CO regulate pyroptosis.

### 7.3. HO-1 and Regulated Necrosis (Necroptosis)

Necroptosis, a genetically-regulated form of necrotic cell death, has emerging significance in human disease [193,194,195,196]. Necroptosis presents many of the morphological features of accidental necrosis including organelle swelling, plasma membrane rupture, cell lysis and leakage of intracellular components, which in turn may propagate secondary inflammatory responses via release of damage-associated molecular patterns (DAMPs) [16,197]. Thus, similar to non-regulated necrosis, necroptosis represents an inflammatory mode of cell death [198,199]. The necroptosis pathway responds to diverse signals including cellular stimulation with death-receptor ligands. Necroptosis is regulated by receptor-interacting protein kinases-1 and -3 (RIPK1, RIPK3), and mixed-lineage kinase domain-like pseudokinase (MLKL), which in the canonical pathway oligomerize to form a regulatory “*necrosome*” complex [200,201]. The phosphorylation of MLKL by RIPK3 is the primary event in necroptosis activation [200,201].

HO-1 has been associated with cytoprotection against both apoptotic and necrotic cell death, in a dose-dependent and cell type dependent fashion. It is plausible that this protection would extend to regulated forms of necrosis (necroptosis). To date, however, only a few studies have examined the relationship between HO-1 and necroptosis in cellular and injury models. Free heme is a cytotoxic agent, which can act as a pro-oxidant, via its central iron chelate. Heme released from hemoglobin can injury endothelial cells of the vasculature by causing membrane damage. Pro-inflammatory effects of heme have been implicated in the pathogenesis of sepsis and malaria. In vitro experiments showed that heme treatment of macrophages can cause macrophage cell death with morphological features of necrosis. The authors found that heme-induced necrotic cell death was dependent on TLR4 regulated TNF production and enhanced ROS generation. Applications of antioxidants and JNK inhibitors or of necrostatin-1, a selective inhibitor of receptor-interacting protein 1 (RIPK1) was protective. Similarly, cells genetically deficient in *Ripk1* or *Ripk3* were protected from heme-induced cell death. Macrophages from *Hmox1*^−/−^ mice were also more sensitive to heme toxicity and oxidative stress [202].

In a model of hepatosteatosis induced by a high-fat diet in mice, RIPK3 was shown to mediate hepatic injury mice, and conversely genetic deficiency in RIPK3 improved the phenotype by reducing oxidative stress and the NF-κB-dependent inflammatory response and activating the Nrf2/HO-1 axis. RIPK3 also mediated TLR4-dependent inflammation in palmitate or LPS activated hepatocytes. Interference with the Nrf2/HO-1 axis reversed the protective effect of RIPK3 deletion in LPS or palmitate exposed hepatocytes [203].

In contrast, this relationship was reversed in a model of post-hepatic I/R-mediated metastasis of colorectal cancer. RIPK3 was responsible for Kuppfer cell death and promoting metastasis following I/R in this model, in conjunction with elevated macrophage TNF and HO-1 production which were immunosuppressive. Deficiency of TNF promoted tumor progression whereas conversely, a monocyte/macrophage-specific deficiency in HO-1 or inhibition of HO-1 reversed the immunosuppressive effect of macrophages, and reduced tumor progression post-I/R. The authors concluded that host cell RIPK3 deficiency suppressed HO-1 expression level and was associated with reduced immune cell recruitment and inhibition of the tumor outgrowth [204].

In a model of oxidant-induced mixed cardiomyocyte cell death with features of both apoptosis and necroptosis, preconditioning with dexmedetomidine (Dex), an α2-adrenoceptor (α2-AR) agonist, resulted in reciprocal upregulation of HO-1 and downregulation of RIPK1/RIPK3 [205]. Taken together, these intriguing examples suggest a regulatory relationship between HO-1 and RIPK3-dependent necroptosis, with heme removal as a possible mechanism for HO-1-mediated cytoprotection.

## 8. HO-1, a Mediator of Ferroptosis

Ferroptosis is defined as a uniquely iron-dependent necrotic form of necrotic cell death that is distinct from autophagy, apoptosis and other forms of necrosis-like RCD, including necroptosis, and which has been implicated in the propagation of inflammation [206,207]. The morphologically distinct features of ferroptosis include mitochondrial shrinkage and increased mitochondrial membrane density [206]. Blockage of cystine uptake is a primary stimulator of ferroptosis, which results in impaired synthesis of reduced glutathione (GSH) for use as substrate for (phospholipid-hydroperoxide) glutathione peroxidase-4 (GPX4)-mediated detoxification of organic hydroperoxides [208]. Iron can catalyze the peroxidation of lipids resulting in membrane disruption characteristic of ferroptosis. Inhibition of cystine uptake also promotes the degradation of ferritin via a nuclear receptor coactivator 4 (NCOA4)-mediated selective autophagy mechanism [209,210]. Ferroptotic cell death is inhibited by lipophilic antioxidants, such as ferrostatin-1 and others [211]; and by iron chelators [212], which also can contextually inhibit oxidant stimulated HO-1 expression [213,214]. Although HO-1 is generally found to be protective in autophagy and other types of RCD, its specific role in ferroptosis remains unclear. While HO-1 may be protective in mitigating pro-oxidant states by preserving mitochondrial function, as implicated in the initiation of inflammatory cell death and apoptosis, HO-1 releases iron as a reaction by-product, which is thereby implicated in the initiation of ferroptosis if in excess or left unsequestered by ferritin. Thus, whether HO-1 is regarded as a promoter or inhibitor of ferroptosis is context-dependent and varies with model studies.

Ferroptosis is implicated as a pathogenic mechanism of I/R or doxorubicin-mediated cardiomyopathy. HO-1 increased in heart tissue following doxorubicin challenge and was associated with iron deposition in cardiac mitochondria. The heme oxygenase inhibitor zinc-protoporphyrin-IX (ZnPP), as well as mitochondria-targeted antioxidant, were found to be protective in this model. Furthermore, ferroptosis-associated cardiomyocyte injury could be alleviated by iron chelators and ferrostatin [215]. Downregulation of the iron exported feroportin worsened ferroptosis-related injury in a model of intracerebral hemorrhage [216]. These results indicate that HO-1, and specifically HO-1-derived iron, can represent a pathogenic player in cardiomyopathy via iron-dependent ferroptosis and may represent a therapeutic target for inhibition in this context.

The oncogenic RAS-selective lethal small molecule erastin promotes ferroptosis. HO-1 was found to be a critical mediator of erastin-induced ferroptosis in cancer cells, as confirmed by genetic validation studies [217]. ZnPP, a HO-1 inhibitor, prevented erastin-induced ferroptotic cell death, whereas HO-1 induction mediated by reagents such as heme or CORM, promoted cell death in this model. These results affirmed the critical role for HO-1 in promoting cancer cell ferroptosis [217]. Treatment with BAY 11-7085 (BAY), an I-κBα inhibitor, induced ferroptotic death in cancer cells. In this model, ferropoptosis was associated with increased Nrf2-dependent HO-1 expression, with subsequent mitochondrial and nuclear translocation of HO-1 and increased mitochondrial dysfunction and mitophagy-dependent turnover [218].

In contrast, some studies have implicated HO-1 as a cytoprotective mechanism against ferroptosis as the product of induction by pre-conditioning agents. Kidney injury during rhabdomyolysis was associated with ferroptosis as it was shown to be inhibited by ferrostatin but not sensitive to RIPK3 deletion [219]. The antioxidant curcumin was shown to reduce kidney injury during rhabdomyolysis via downregulation of ferroptosis. Curcumin is a potent inducer of HO-1 in the kidney, and HO-1 was implicated in the protective effects [219]. An antiferroptotic role for HO-1 was also proposed in kidney epithelial cells subjected to erastin. In this model, *Hmox1*^−/−^ kidney proximal tubule epithelial cells were sensitized to erastin-mediated ferroptosis [220]. These intriguing experiments suggest that HO-1 plays a complex role in ferroptosis that required further experimental clarification.

## 9. Conclusions

HO-1 has emerged from its canonical role as a metabolic enzyme primarily engaged in hemoprotein turnover, to a pleiotropic mediator of cellular function with potential impact on inflammatory processes [1,2,14].The mechanisms by which HO-1 may impact cellular processes are multivariate, and traditionally have been related to its enzymatic activity and the generation of its reaction products, BV, iron and CO [2]. Beneficial functions of HO-1 in cellular regulation, and also of its reaction product CO, largely involve modulation of apoptosis, inflammation and cell proliferation [2,221]. HO-1 may serve compartment-specific roles, including potential activity-dependent roles in the caveolae, and mitochondria [126,127]. Emerging studies also suggest the existence of non-canonical roles of HO-1 in effectuating cellular function, which are independent of heme catabolic activity [121,122]. Of these include a nuclear form of the protein that regulates nuclear transcriptional activity [124]. HO-1 has an intricate and incompletely understood role in autophagy regulation and may positively or negatively coordinate with this process in the maintenance of cellular defenses [222]. HO-1 also impacts the outcome of genetically regulated cell death programs including apoptosis and other forms of necrosis-like RCD. While HO-1 is generally associated with cytoprotection and inhibition of cell death processes, in the case of ferroptosis, HO-1 may alternately serve to aggravate this process in a context-dependent fashion.

These observations underscore the need for HO-1 to be tightly regulated to achieve cytoprotective effects. HO-1 remains an attractive candidate therapeutic target for remediation of inflammatory and other diseases [14]. Strategies to harness this therapeutic role have included preconditioning with natural antioxidants and other inducers of HO-1, *Hmox1* gene therapy approaches [223,224], and application of the end products of HO activity [14,114,225]. Most notably, CO, has been proposed as a therapeutic mimic of HO-1, as achieved through the application of the gas or by pharmacological administration of chemical donor compounds, including transition metal containing carbon monoxide releasing molecules CORMs and organic CO donors [114,226,227,228,229]. Harnessing the therapeutic potential role of HO-1 will depend on a comprehensive understanding of its context-dependent impact, both positive and negative, on cell survival and death mechanisms, as discussed in this review, including multimodal cell death involving or dependent on autophagy, pyroptosis, necroptosis, and ferroptosis pathways. This is especially important, as the role of these processes themselves may have context-dependent and variable roles in the propagation of inflammation and disease. Further, the roles of HO-1 in either mitigating or amplifying these processes in the progression of human disease are only partially understood, and some such as autophagy and necroptosis may have both protective and harmful sequelae.

## Data Availability

N/A.

## References

[1] Abraham N.G., Kappas A. (2008). Pharmacological and clinical aspects of heme oxygenase. Pharmacol. Rev..

[2] Ryter S.W., Alam J., Choi A.M. (2006). Heme oxygenase-1/carbon monoxide: From basic science to therapeutic applications. Physiol. Rev..

[3] Tenhunen R., Marver H.S., Schmid R. (1969). Microsomal heme oxygenase. Characterization of the enzyme. J. Biol. Chem..

[4] Tenhunen R., Ross M.E., Marver H.S., Schmid R. (1970). Reduced nicotinamide-adenine dinucleotide phosphate dependent biliverdin reductase: Partial purification and characterization. Biochemistry.

[5] Maines M.D. (1997). The heme oxygenase system: A regulator of second messenger gases. Annu. Rev. Pharmacol. Toxicol..

[6] Cruse I., Maines M.D. (1988). Evidence suggesting that the two forms of heme oxygenase are products of different genes. J. Biol. Chem..

[7] Keyse S.M., Tyrrell R.M. (1989). Heme oxygenase is the major 32-kDa stress protein induced in human skin fibroblasts by UVA radiation, hydrogen peroxide, and sodium arsenite. Proc. Natl. Acad. Sci. USA.

[8] Shibahara S., Müller R., Taguchi H., Yoshida T. (1985). Cloning and expression of cDNA for rat heme oxygenase. Proc. Natl. Acad. Sci. USA.

[9] Alam J., Shibahara S., Smith A. (1989). Transcriptional activation of the heme oxygenase gene by heme and cadmium in mouse hepatoma cells. J. Biol. Chem..

[10] Poss K.D., Tonegawa S. (1997). Heme oxygenase-1 is required for mammalian iron reutilization. Proc. Natl. Acad. Sci. USA.

[11] Poss K.D., Tonegawa S. (1997). Reduced stress defense in heme oxygenase 1-deficient cells. Proc. Natl. Acad. Sci. USA.

[12] Yachie A., Niida Y., Wada T., Igarashi N., Kaneda H., Toma T., Ohta K., Kasahara Y., Koizumi S. (1999). Oxidative stress causes enhanced endothelial cell injury in human heme oxygenase-1 deficiency. J Clin. Invest..

[13] Otterbein L.E., Bach F.H., Alam J., Soares M., Tao Lu H., Wysk M., Davis R.J., Flavell R.A., Choi A.M. (2000). Carbon monoxide has anti-inflammatory effects involving the mitogen-activated protein kinase pathway. Nat. Med..

[14] Ryter S.W., Choi A.M.K. (2016). Targeting heme oxygenase-1 and carbon monoxide for therapeutic modulation of inflammation. Transl. Res..

[15] Brouard S., Otterbein L.E., Anrather J., Tobiasch E., Bach F.H., Choi A.M., Soares M.P. (2000). Carbon monoxide generated by heme oxygenase-1 suppresses endothelial cell apoptosis. J. Exp. Med..

[16] Galluzzi L., Vitale I., Aaronson S.A., Abrams J.M., Adam D., Agostinis P., Alnemri E.S., Altucci L., Amelio I., Andrews D.W. (2018). Molecular mechanisms of cell death: Recommendations of the Nomenclature Committee on Cell Death 2018. Cell Death Differ..

[17] Galluzzi L., Maiuri M.C., Vitale I., Zischka H., Castedo M., Zitvogel L., Kroemer G. (2007). Cell death modalities: Classification and pathophysiological implications. Cell Death Differ..

[18] Majno G., Joris I. (1995). Apoptosis, oncosis, and necrosis. An overview of cell death. Am. J Pathol..

[19] Kroemer G., Dallaporta B., Resche-Rigon M. (1998). The mitochondrial death/life regulator in apoptosis and necrosis. Annu. Rev. Physiol..

[20] Kist M., Vucic D. (2021). Cell death pathways: Intricate connections and disease implications. EMBO J..

[21] Galluzzi L., Baehrecke E.H., Ballabio A., Boya P., Bravo-San Pedro J.M., Cecconi F., Choi A.M., Chu C.T., Codogno P., Colombo M.I. (2017). Molecular definitions of autophagy and related processes. EMBO J..

[22] Pittala V., Vanella L., Salerno L., Romeo G., Marrazzo A., Di Giacomo C., Sorrenti V. (2018). Effects of polyphenolic derivatives on heme oxygenase-system in metabolic dysfunctions. Curr. Med. Chem..

[23] Hahn D., Shin S.H., Bae J.S. (2020). Natural antioxidant and anti-inflammatory compounds in foodstuff or medicinal herbs inducing heme oxygenase-1 expression. Antioxidants.

[24] Bajpai V.K., Alam M.B., Ju M.K., Kwon K.R., Huh Y.S., Han Y.K., Lee S.H. (2018). Antioxidant mechanism of polyphenol-rich Nymphaea nouchali leaf extract protecting DNA damage and attenuating oxidative stress-induced cell death via Nrf2-mediated heme-oxygenase-1 induction coupled with ERK/p38 signaling pathway. Biomed. Pharmacother..

[25] Scapagnini G., Foresti R., Calabrese V., Giuffrida Stella A.M., Green C.J., Motterlini R. (2002). Caffeic acid phenethyl ester and curcumin: A novel class of heme oxygenase-1 inducers. Mol. Pharmacol..

[26] Martin D., Rojo A.I., Salinas M., Diaz R., Gallardo G., Alam J., De Galarreta C.M., Cuadrado A. (2004). Regulation of heme oxygenase-1 expression through the phosphatidylinositol 3-kinase/Akt pathway and the Nrf2 transcription factor in response to the antioxidant phytochemical carnosol. J. Biol. Chem..

[27] Foresti R., Hoque M., Monti D., Green C.J., Motterlini R. (2005). Differential activation of heme oxygenase-1 by chalcones and rosolic acid in endothelial cells. J. Pharmacol. Exp. Ther..

[28] Ogborne R.M., Rushworth S.A., Charalambos C., O’Connell M.A. (2004). Haem oxygenase-1: A target for dietary antioxidants. Biochem. Soc. Trans..

[29] Lee P.J., Alam J., Sylvester S.L., Inamdar N., Otterbein L., Choi A.M. (1996). Regulation of heme oxygenase-1 expression in vivo and in vitro in hyperoxic lung injury. Am. J. Respir. Cell Mol. Biol..

[30] Lee P.J., Jiang B.H., Chin B.Y., Iyer N.V., Alam J., Semenza G.L., Choi A.M. (1997). Hypoxia-inducible factor-1 mediates transcriptional activation of the heme oxygenase-1 gene in response to hypoxia. J. Biol. Chem..

[31] Kitamuro T., Takahashi K., Ogawa K., Udono-Fujimori R., Takeda K., Furuyama K., Nakayama M., Sun J., Fujita H., Hida W. (2003). Bach1 functions as a hypoxia-inducible repressor for the heme oxygenase-1 gene in human cells. J. Biol. Chem..

[32] Keyse S.M., Applegate L.A., Tromvoukis Y., Tyrrell R.M. (1990). Oxidant stress leads to transcriptional activation of the human heme oxygenase gene in cultured skin fibroblasts. Mol. Cell Biol..

[33] Alam J., Stewart D., Touchard C., Boinapally S., Choi A.M., Cook J.L. (1999). Nrf2, a Cap’n’Collar transcription factor, regulates induction of the heme oxygenase-1 gene. J. Biol. Chem..

[34] Kang M.I., Kobayashi A., Wakabayashi N., Kim S.G., Yamamoto M. (2004). Scaffolding of Keap1 to the actin cytoskeleton controls the function of Nrf2 as key regulator of cytoprotective phase 2 genes. Proc. Natl. Acad. Sci. USA.

[35] Zipper L.M., Mulcahy R.T. (2002). The Keap1 BTB/POZ dimerization function is required to sequester Nrf2 in cytoplasm. J. Biol. Chem..

[36] Itoh K., Wakabayashi N., Katoh Y., Ishii T., O’Connor T., Yamamoto M. (2003). Keap1 regulates both cytoplasmic-nuclear shuttling and degradation of Nrf2 in response to electrophiles. Genes Cells.

[37] Itoh K., Wakabayashi N., Katoh Y., Ishii T., Igarashi K., Engel J.D., Yamamoto M. (1999). Keap1 represses nuclear activation of antioxidant responsive elements by Nrf2 through binding to the amino-terminal Neh2 domain. Genes Dev..

[38] Igarashi K., Sun J. (2006). The heme-Bach1 pathway in the regulation of oxidative stress response and erythroid differentiation. Antioxid. Redox Signal..

[39] Sun J., Hoshino H., Takaku K., Nakajima O., Muto A., Suzuki H., Tashiro S., Takahashi S., Shibahara S., Alam J. (2002). Hemoprotein Bach1 regulates enhancer availability of heme oxygenase-1 gene. EMBO J..

[40] Oyake T., Itoh K., Motohashi H., Hayashi N., Hoshino H., Nishizawa M., Yamamoto M., Igarashi K. (1996). Bach proteins belong to a novel family of BTB-basic leucine zipper transcription factors that interact with MafK and regulate transcription through the NF-E2 site. Mol. Cell Biol..

[41] Sun J., Brand M., Zenke Y., Tashiro S., Groudine M., Igarashi K. (2004). Heme regulates the dynamic exchange of Bach1 and NF-E2-related factors in the Maf transcription factor network. Proc. Natl. Acad. Sci. USA.

[42] Alam J., Killeen E., Gong P., Naquin R., Hu B., Stewart D., Ingelfinger J.R., Nath K.A. (2003). Heme activates the heme oxygenase-1 gene in renal epithelial cells by stabilizing Nrf2. Am. J. Physiol. Renal Physiol..

[43] Alam J., Cai J., Smith A. (1994). Isolation and characterization of the mouse heme oxygenase-1 gene. Distal 5’ sequences are required for induction by heme or heavy metals. J. Biol. Chem..

[44] Alam J., Camhi S., Choi A.M. (1995). Identification of a second region upstream of the mouse heme oxygenase-1 gene that functions as a basal level and inducer-dependent transcription enhancer. J. Biol. Chem..

[45] Inamdar N.M., Ahn Y.I., Alam J. (1996). The heme-responsive element of the mouse heme oxygenase-1 gene is an extended AP-1 binding site that resembles the recognition sequences for MAF and NF-E2 transcription factors. Biochem. Biophys. Res. Commun..

[46] Alam J., Cook J.L. (2007). How many transcription factors does it take to turn on the heme oxygenase-1 gene?. Am. J. Respir. Cell Mol. Biol..

[47] Alam J., Igarashi K., Immenschuh S., Shibahara S., Tyrrell R.M. (2004). Regulation of heme oxygenase-1 gene transcription: Recent advances and highlights from the International Conference (Uppsala, 2003) on Heme Oxygenase. Antioxid. Redox Signal..

[48] Morse D., Lin L., Choi A.M., Ryter S.W. (2009). Heme oxygenase-1, a critical arbitrator of cell death pathways in lung injury and disease. Free Radic. Biol. Med..

[49] Medina M.V., Sapochnik D., Garcia Solá M., Coso O. (2020). Regulation of the expression of heme oxygenase-1: Signal transduction, gene promoter activation, and beyond. Antioxid. Redox Signal..

[50] Piras S., Furfaro A.L., Caggiano R., Brondolo L., Garibaldi S., Ivaldo C., Marinari U.M., Pronzato M.A., Faraonio R., Nitti M. (2018). microRNA-494 Favors HO-1 Expression in neuroblastoma cells exposed to oxidative stress in a Bach1-independent way. Front. Oncol..

[51] Skrzypek K., Tertil M., Golda S., Ciesla M., Weglarczyk K., Collet G., Guichard A., Kozakowska M., Boczkowski J., Was H. (2013). Interplay between heme oxygenase-1 and miR-378 affects non-small cell lung carcinoma growth, vascularization, and metastasis. Antioxid. Redox Signal..

[52] Xiao S., Wang X., Ni H., Li N., Zhang A., Liu H., Pu F., Xu L., Gao J., Zhao Q. (2015). MiR-24-3p promotes porcine reproductive and respiratory syndrome virus replication through suppression of heme oxygenase-1 expression. J. Virol..

[53] Lorenzen J.M., Kaucsar T., Schauerte C., Schmitt R., Rong S., Hübner A., Scherf K., Fiedler J., Martino F., Kumarswamy R. (2014). MicroRNA-24 antagonism prevents renal ischemia reperfusion injury. J. Am. Soc. Nephrol..

[54] Fiedler J., Stöhr A., Gupta S.K., Hartmann D., Holzmann A., Just A., Hansen A., Hilfiker-Kleiner D., Eschenhagen T., Thum T. (2014). Functional microRNA library screening identifies the hypoxamir miR-24 as a potent regulator of smooth muscle cell proliferation and vascularization. Antioxid. Redox Signal..

[55] Stachurska A., Ciesla M., Kozakowska M., Wolffram S., Boesch-Saadatmandi C., Rimbach G., Jozkowicz A., Dulak J., Loboda A. (2013). Cross-talk between microRNAs, nuclear factor E2-related factor 2, and heme oxygenase-1 in ochratoxin A-induced toxic effects in renal proximal tubular epithelial cells. Mol. Nutr. Food Res..

[56] Zhang J., Vandevenne P., Hamdi H., Van Puyvelde M., Zucchi A., Bettonville M., Weatherly K., Braun M.Y. (2015). Micro-RNA-155-mediated control of heme oxygenase 1 (HO-1) is required for restoring adaptively tolerant CD4^+^ T-cell function in rodents. Eur. J. Immunol..

[57] Beckman J.D., Chen C., Nguyen J., Thayanithy V., Subramanian S., Steer C.J., Vercellotti G.M. (2011). Regulation of heme oxygenase-1 protein expression by miR-377 in combination with miR-217. J. Biol. Chem..

[58] Chen L., Zhong J.L. (2020). MicroRNA and heme oxygenase-1 in allergic disease. Int. Immunopharmacol..

[59] Wang Y., Song Y., Pang Y., Yu Z., Hua W., Gu Y., Qi J., Wu H. (2020). miR-183-5p alleviates early injury after intracerebral hemorrhage by inhibiting heme oxygenase-1 expression. Aging.

[60] Cheng X., Ku C.H., Siow R.C. (2013). Regulation of the Nrf2 antioxidant pathway by microRNAs: New players in micromanaging redox homeostasis. Free Radic. Biol. Med..

[61] Narasimhan M., Patel D., Vedpathak D., Rathinam M., Henderson G., Mahimainathan L. (2012). Identification of novel microRNAs in post-transcriptional control of Nrf2 expression and redox homeostasis in neuronal, SH-SY5Y cells. PLoS ONE.

[62] Kim J.H., Lee K.S., Lee D.K., Kim J., Kwak S.N., Ha K.S., Choe J., Won M.H., Cho B.R., Jeoung D. (2014). Hypoxia-responsive MicroRNA-101 promotes angiogenesis via heme oxygenase-1/Vascular endothelial growth factor axis by targeting Cullin 3. Antioxid. Redox Signal..

[63] Do M.T., Kim H.G., Choi J.H., Jeong H.G. (2014). Metformin induces microRNA-34a to downregulate the Sirt1/Pgc-1α/Nrf2 pathway, leading to increased susceptibility of wild-type p53 cancer cells to oxidative stress and therapeutic agents. Free Radic. Biol. Med..

[64] Hou W., Zhu X., Liu J., Ma J. (2020). Inhibition of miR-153 ameliorates ischemia/reperfusion-induced cardiomyocytes apoptosis by regulating Nrf2/HO-1 signaling in rats. Biomed. Eng. Online.

[65] Eades G., Yang M., Yao Y., Zhang Y., Zhou Q. (2011). miR-200a regulates Nrf2 activation by targeting Keap1 mRNA in breast cancer cells. J. Biol. Chem..

[66] Zhang C., Kong X., Ma D. (2020). miR-141-3p inhibits vascular smooth muscle cell proliferation and migration via regulating Keap1/Nrf2/HO-1 pathway. IUBMB Life..

[67] Pulkkinen K.H., Ylä-Herttuala S., Levonen A.L. (2011). Heme oxygenase 1 is induced by miR-155 via reduced BACH1 translation in endothelial cells. Free Radic. Biol. Med..

[68] Hou W., Tian Q., Zheng J., Bonkovsky H.L. (2010). MicroRNA-196 represses Bach1 protein and hepatitis C virus gene expression in human hepatoma cells expressing hepatitis C viral proteins. Hepatology.

[69] Hou W., Tian Q., Steuerwald N.M., Schrum L.W., Bonkovsky H.L. (2012). The let-7 microRNA enhances heme oxygenase-1 by suppressing Bach1 and attenuates oxidant injury in human hepatocytes. Biochim. Biophys. Acta.

[70] Sun X., Li X., Ma S., Guo Y., Li Y. (2018). MicroRNA-98-5p ameliorates oxygen-glucose deprivation/reoxygenation (OGD/R)-induced neuronal injury by inhibiting Bach1 and promoting Nrf2/ARE signaling. Biochem. Biophys. Res. Commun..

[71] Kozakowska M., Szade K., Dulak J., Jozkowicz A. (2014). Role of heme oxygenase-1 in postnatal differentiation of stem cells: A possible cross-talk with microRNAs. Antioxid. Redox Signal..

[72] Grochot-Przeczek A., Dulak J., Jozkowicz A. (2012). Haem oxygenase-1: Non-canonical roles in physiology and pathology. Clin. Sci..

[73] Kozakowska M., Ciesla M., Stefanska A., Skrzypek K., Was H., Jazwa A., Grochot-Przeczek A., Kotlinowski J., Szymula A., Bartelik A. (2012). Heme oxygenase-1 inhibits myoblast differentiation by targeting myomirs. Antioxid. Redox Signal..

[74] Yu D., Sun R., Shen D., Ge L., Xue T., Cao Y. (2020). Nuclear heme oxygenase-1 improved the hypoxia-mediated dysfunction of blood-spinal cord barrier via the miR-181c-5p/SOX5 signaling pathway. Neuroreport.

[75] Ciesla M., Marona P., Kozakowska M., Jez M., Seczynska M., Loboda A., Bukowska-Strakova K., Szade A., Walawender M., Kusior M. (2016). Heme oxygenase-1 controls an HDAC4-miR-206 pathway of oxidative stress in rhabdomyosarcoma. Cancer Res..

[76] Yamada N., Yamaya M., Okinaga S., Nakayama K., Sekizawa K., Shibahara S., Sasaki H. (2000). Microsatellite polymorphism in the heme oxygenase-1 gene promoter is associated with susceptibility to emphysema. Am. J. Hum. Genet..

[77] Pechlaner R., Willeit P., Summerer M., Santer P., Egger G., Kronenberg F., Demetz E., Weiss G., Tsimikas S., Witztum J.L. (2015). Heme oxygenase-1 gene promoter microsatellite polymorphism is associated with progressive atherosclerosis and incident cardiovascular disease. Arterioscler. Thromb. Vasc. Biol..

[78] Liang K.W., Lee W.J., Lee W.L., Wu J.P., Lee I.T., Wang J.S., Sheu W.H. (2021). Subjects with coronary artery disease and reduced ejection fraction have longer (GT)n repeats in the heme-oxygenase 1 gene promoter. Heart Vessel..

[79] Chen Y.H., Chau L.Y., Chen J.W., Lin S.J. (2008). Serum bilirubin and ferritin levels link heme oxygenase-1 gene promoter polymorphism and susceptibility to coronary artery disease in diabetic patients. Diabetes Care..

[80] Sheu C.C., Zhai R., Wang Z., Gong M.N., Tejera P., Chen F., Su L., Thompson B.T., Christiani D.C. (2009). Heme oxygenase-1 microsatellite polymorphism and haplotypes are associated with the development of acute respiratory distress syndrome. Intensive Care Med..

[81] Vilander L.M., Vaara S.T., Donner K.M., Lakkisto P., Kaunisto M.A., Pettilä V., FINNAKI Study Group (2019). Heme oxygenase-1 repeat polymorphism in septic acute kidney injury. PLoS ONE..

[82] Kaartokallio T., Klemetti M.M., Timonen A., Uotila J., Heinonen S., Kajantie E., Kere J., Kivinen K., Pouta A., Lakkisto P. (2014). Microsatellite polymorphism in the heme oxygenase-1 promoter is associated with nonsevere and late-onset preeclampsia. Hypertension.

[83] Bao W., Song F., Li X., Rong S., Yang W., Wang D., Xu J., Fu J., Zhao Y., Liu L. (2010). Association between heme oxygenase-1 gene promoter polymorphisms and type 2 diabetes mellitus: A HuGE review and meta-analysis. Am. J. Epidemiol..

[84] Du Y., Zhang H., Xu Y., Ding Y., Chen X., Mei Z., Ding H., Jie Z. (2019). Association among genetic polymorphisms of GSTP1, HO-1, and SOD-3 and chronic obstructive pulmonary disease susceptibility. Int. J. Chron. Obstruct. Pulmon. Dis..

[85] Zhou H., Ying X., Liu Y., Ye S., Yan J., Li Y. (2017). Genetic polymorphism of heme oxygenase 1 promoter in the occurrence and severity of chronic obstructive pulmonary disease: A meta-analysis. J. Cell Mol. Med..

[86] Fu W.P., Zhao Z.H., Fang L.Z., Sun C., Liu L., Zhang J.Q., Zhang Y.P., Dai L.M. (2007). Heme oxygenase-1 polymorphism associated with severity of chronic obstructive pulmonary disease. Chin. Med. J..

[87] Zhang J.Q., Zhang J.Q., Fang L.Z., Liu L., Fu W.P., Dai L.M. (2015). Effect of oral N-acetylcysteine on COPD patients with microsatellite polymorphism in the heme oxygenase-1 gene promoter. Drug Des. Devel. Ther..

[88] Nakayama K., Kikuchi A., Yasuda H., Ebihara S., Sasaki T., Ebihara T., Yamaya M. (2006). Heme oxygenase-1 gene promoter polymorphism and decline in lung function in Japanese men. Thorax.

[89] Guenegou A., Leynaert B., Benessiano J., Pin I., Demoly P., Neukirch F., Boczkowski J., Aubier M. (2006). Association of lung function decline with the heme oxygenase-1 gene promoter microsatellite polymorphism in a general population sample. Results from the European Community Respiratory Health Survey (ECRHS), France. J. Med. Genet..

[90] He J.Q., Ruan J., Connett J.E., Anthonisen N.R., Pare P.D., Sandford A.J. (2002). Antioxidant gene polymorphisms and susceptibility to a rapid decline in lung function in smokers. Am. J. Respir. Crit. Care Med..

[91] Hersh C.P., Demeo D.L., Lange C., Litonjua A.A., Reilly J.J., Kwiatkowski D., Laird N., Sylvia J.S., Sparrow D., Speizer F.E. (2005). Attempted replication of reported chronic obstructive pulmonary disease candidate gene associations. Am. J. Respir. Cell Mol. Biol..

[92] Hirai H., Kubo H., Yamaya M., Nakayama K., Numasaki M., Kobayashi S., Suzuki S., Shibahara S., Sasaki H. (2003). Microsatellite polymorphism in heme oxygenase-1 gene promoter is associated with susceptibility to oxidant-induced apoptosis in lymphoblastoid cell lines. Blood.

[93] Haines D.D., Tosaki A. (2020). Heme degradation in pathophysiology of and countermeasures to inflammation-associated disease. Int. J. Mol. Sci..

[94] Ryter S.W., Tyrrell R.M. (2000). The heme synthesis and degradation pathways: Role in oxidant sensitivity. Heme oxygenase has both pro- and antioxidant properties. Free Radic. Biol. Med..

[95] Immenschuh S., Vijayan V., Janciauskiene S., Gueler F. (2017). Heme as a target for therapeutic interventions. Front. Pharmacol..

[96] Donegan R.K., Moore C.M., Hanna D.A., Reddi A.R. (2019). Handling heme: The mechanisms underlying the movement of heme within and between cells. Free Radic. Biol. Med..

[97] Kumar A., Ganini D., Deterding L.J., Ehrenshaft M., Chatterjee S., Mason R.P. (2013). Immuno-spin trapping of heme-induced protein radicals: Implications for heme oxygenase-1 induction and heme degradation. Free Radic. Biol. Med..

[98] Jeney V., Balla J., Yachie A., Varga Z., Vercellotti G.M., Eaton J.W., Balla G. (2002). Pro-oxidant and cytotoxic effects of circulating heme. Blood.

[99] Balla J., Jacob H.S., Balla G., Nath K., Eaton J.W., Vercellotti G.M. (1993). Endothelial-cell heme uptake from heme proteins: Induction of sensitization and desensitization to oxidant damage. Proc. Natl. Acad. Sci. USA.

[100] Larsen R., Gozzelino R., Jeney V., Tokaji L., Bozza F.A., Japiassú A.M., Bonaparte D., Cavalcante M.M., Chora A., Ferreira A. (2010). A central role for free heme in the pathogenesis of severe sepsis. Sci. Transl. Med..

[101] Pereira M.L.M., Marinho C.R.F., Epiphanio S. (2018). Could heme oxygenase-1 be a new target for therapeutic intervention in malaria-associated acute lung injury/acute respiratory distress syndrome?. Front. Cell Infect. Microbiol..

[102] Halliwell B., Gutteridge J.M. (1984). Oxygen toxicity, oxygen radicals, transition metals and disease. Biochem. J..

[103] Dröge W. (2002). Free radicals in the physiological control of cell function. Physiol. Rev..

[104] Vile G.F., Tyrrell R.M. (1993). Oxidative stress resulting from ultraviolet A irradiation of human skin fibroblasts leads to a heme oxygenase-dependent increase in ferritin. J. Biol. Chem..

[105] Vile G.F., Basu-Modak S., Waltner C., Tyrrell R.M. (1994). Heme oxygenase 1 mediates an adaptive response to oxidative stress in human skin fibroblasts. Proc. Natl. Acad. Sci. USA.

[106] Arosio P., Levi S. (2002). Ferritin, iron homeostasis, and oxidative damage. Free Radic. Biol. Med..

[107] Balla G., Jacob H.S., Balla J., Rosenberg M., Nath K., Apple F., Eaton J.W., Vercellotti G.M. (1992). Ferritin: A cytoprotective antioxidant strategem of endothelium. J. Biol. Chem..

[108] Juckett M.B., Balla J., Balla G., Jessurun J., Jacob H.S., Vercellotti G.M. (1995). Ferritin protects endothelial cells from oxidized low density lipoprotein in vitro. Am. J. Pathol..

[109] Balla J., Nath K.A., Balla G., Juckett M.B., Jacob H.S., Vercellotti G.M. (1995). Endothelial cell heme oxygenase and ferritin induction in rat lung by hemoglobin in vivo. Am. J. Physiol..

[110] Stocker R., Yamamoto Y., McDonagh A.F., Glazer A.N., Ames B.N. (1987). Bilirubin is an antioxidant of possible physiological importance. Science.

[111] Neuzil J., Stocker R. (1993). Bilirubin attenuates radical-mediated damage to serum albumin. FEBS Lett..

[112] Stocker R., Glazer A.N., Ames B.N. (1987). Antioxidant activity of albumin-bound bilirubin. Proc. Natl. Acad. Sci USA.

[113] Stocker R., Ames B.N. (1987). Potential role of conjugated bilirubin and copper in the metabolism of lipid peroxides in bile. Proc. Natl. Acad. Sci USA.

[114] Motterlini R., Otterbein L.E. (2010). The therapeutic potential of carbon monoxide. Nat. Rev. Drug Discov..

[115] Ryter S.W., Ma K.C., Choi A.M.K. (2018). Carbon monoxide in lung cell physiology and disease. Am. J. Physiol. Cell Physiol..

[116] Otterbein L.E., Choi A.M. (2000). Heme oxygenase: Colors of defense against cellular stress. Am. J. Physiol. Lung Cell. Mol. Physiol..

[117] Kvam E., Hejmadi V., Ryter S., Pourzand C., Tyrrell R.M. (2000). Heme oxygenase activity causes transient hypersensitivity to oxidative ultraviolet A radiation that depends on release of iron from heme. Free Radic. Biol. Med..

[118] Suttner D.M., Dennery P.A. (1999). Reversal of HO-1 related cytoprotection with increased expression is due to reactive iron. FASEB J..

[119] Schipper H.M., Song W., Tavitian A., Cressatti M. (2019). The sinister face of heme oxygenase-1 in brain aging and disease. Prog. Neurobiol..

[120] Vanella L., Barbagallo I., Tibullo D., Forte S., Zappalà A., Li Volti G. (2016). The non-canonical functions of the heme oxygenases. Oncotarget.

[121] Dennery P.A. (2014). Signaling function of heme oxygenase proteins. Antioxid Redox Signal..

[122] Wang X., Wang Y., Kim H.P., Nakahira K., Ryter S.W., Choi A.M. (2007). Carbon monoxide protects against hyperoxia-induced endothelial cell apoptosis by inhibiting reactive oxygen species formation. J. Biol. Chem..

[123] Suttner D.M., Sridhar K., Lee C.S., Tomura T., Hansen T.N., Dennery P.A. (1999). Protective effects of transient HO-1 overexpression on susceptibility to oxygen toxicity in lung cells. Am. J. Physiol..

[124] Biswas C., Shah N., Muthu M., La P., Fernando A.P., Sengupta S., Yang G., Dennery P.A. (2014). Nuclear heme oxygenase-1 (HO-1) modulates subcellular distribution and activation of Nrf2, impacting metabolic and anti-oxidant defenses. J. Biol. Chem..

[125] Krzeptowski W., Chudy P., Sokołowski G., Żukowska M., Kusienicka A., Seretny A., Kalita A., Czmoczek A., Gubała J., Baran S. (2021). Proximity ligation assay detection of protein-DNA interactions-is there a link between heme oxygenase-1 and G-quadruplexes?. Antioxidants.

[126] Gray L.T., Puig Lombardi E., Verga D., Nicolas A., Teulade-Fichou M.P., Londoño-Vallejo A., Maizels N. (2019). G-quadruplexes sequester free heme in living cells. Cell Chem. Biol..

[127] Converso D.P., Taillé C., Carreras M.C., Jaitovich A., Poderoso J.J., Boczkowski J. (2006). HO-1 is located in liver mitochondria and modulates mitochondrial heme content and metabolism. FASEB J..

[128] Kim H.P., Wang X., Galbiati F., Ryter S.W., Choi A.M. (2004). Caveolae compartmentalization of heme oxygenase-1 in endothelial cells. FASEB J..

[129] Jung N.H., Kim H.P., Kim B.R., Cha S.H., Kim G.A., Ha H., Na Y.E., Cha Y.N. (2003). Evidence for heme oxygenase-1 association with caveolin-1 and -2 in mouse mesangial cells. IUBMB Life.

[130] Vijayan V., Wagener F.A.D.T.G., Immenschuh S. (2018). The macrophage heme-heme oxygenase-1 system and its role in inflammation. Biochem. Pharmacol..

[131] Willis D., Moore A.R., Frederick R., Willoughby D.A. (1996). Heme oxygenase: A novel target for the modulation of the inflammatory response. Nat. Med..

[132] Inoue S., Suzuki M., Nagashima Y., Suzuki S., Hashiba T., Tsuburai T., Ikehara K., Matsuse T., Ishigatsubo Y. (2001). Transfer of heme oxygenase 1 cDNA by a replication-deficient adenovirus enhances interleukin 10 production from alveolar macrophages that attenuates lipopolysaccharide-induced acute lung injury in mice. Hum. Gene Ther..

[133] Hashiba T., Suzuki M., Nagashima Y., Suzuki S., Inoue S., Tsuburai T., Matsuse T., Ishigatubo Y. (2001). Adenovirus-mediated transfer of heme oxygenase-1 cDNA attenuates severe lung injury induced by the influenza virus in mice. Gene Ther..

[134] Lee T.S., Chau L.Y. (2002). Heme oxygenase-1 mediates the anti-inflammatory effect of interleukin-10 in mice. Nat. Med..

[135] Sarady-Andrews J.K., Liu F., Gallo D., Nakao A., Overhaus M., Ollinger R., Choi A.M., Otterbein L.E. (2005). Biliverdin administration protects against endotoxin-induced acute lung injury in rats. Am. J. Physiol. Lung Cell Mol. Physiol..

[136] Takamiya R., Hung C.C., Hall S.R., Fukunaga K., Nagaishi T., Maeno T., Owen C., Macias A.A., Fredenburgh L.E., Ishizaka A. (2009). High-mobility group box 1 contributes to lethality of endotoxemia in heme oxygenase-1-deficient mice. Am. J. Respir. Cell Mol. Biol..

[137] Tsoyi K., Lee T.Y., Lee Y.S., Kim H.J., Seo H.G., Lee J.H., Chang K.C. (2009). Heme-oxygenase-1 induction and carbon monoxide-releasing molecule inhibit lipopolysaccharide (LPS)-induced high-mobility group box 1 release in vitro and improve survival of mice in LPS- and cecal ligation and puncture-induced sepsis model in vivo. Mol. Pharmacol..

[138] Chung S.W., Liu X., Macias A.A., Baron R.M., Perrella M.A. (2008). Heme oxygenase-1-derived carbon monoxide enhances the host defense response to microbial sepsis in mice. J. Clin. Invest..

[139] Broz P., Dixit V. (2016). Inflammasomes: Mechanism of assembly, regulation and signalling. Nat. Rev. Immunol..

[140] Luo Y.P., Jiang L., Kang K., Fei D.S., Meng X.L., Nan C.C., Pan S.H., Zhao M.R., Zhao M.Y. (2014). Hemin inhibits NLRP3 inflammasome activation in sepsis-induced acute lung injury, involving heme oxygenase-1. Int. Immunopharmacol..

[141] Kim S.J., Lee S.M. (2013). NLRP3 inflammasome activation in D-galactosamine and lipopolysaccharide-induced acute liver failure: Role of heme oxygenase-1. Free Radic. Biol. Med..

[142] Jung S.S., Moon J.S., Xu J., Ifedigbo E., Ryter S.W., Choi A.M., Nakahira K. (2015). Carbon monoxide negatively regulates NLRP3 inflammasome activation in macrophages. Am. J. Physiol. Lung Cell Mol. Physiol..

[143] Wegiel B., Larsen R., Gallo D., Chin B.Y., Harris C., Mannam P., Kaczmarek E., Lee P.J., Zuckerbraun B.S., Flavell R. (2014). Macrophages sense and kill bacteria through carbon monoxide-dependent inflammasome activation. J. Clin. Invest..

[144] Lee K.Y. (2019). M1 and M2 polarization of macrophages: A mini-review. Med. Biol. Sci. Eng..

[145] Naito Y., Takagi T., Higashimura Y. (2014). Heme oxygenase-1 and anti-inflammatory M2 macrophages. Arch. Biochem. Biophys..

[146] Sierra-Filardi E., Vega M.A., Sánchez-Mateos P., Corbí A.L., Puig-Kröger A. (2010). Heme oxygenase-1 expression in M-CSF-polarized M2 macrophages contributes to LPS-induced IL-10 release. Immunobiology.

[147] Zhang M., Nakamura K., Kageyama S., Lawal A.O., Gong K.W., Bhetraratana M., Fujii T., Sulaiman D., Hirao H., Bolisetty S. (2018). Myeloid HO-1 modulates macrophage polarization and protects against ischemia-reperfusion injury. JCI Insight..

[148] Chiang N., Shinohara M., Dalli J., Mirakaj V., Kibi M., Choi A.M., Serhan C.N. (2013). Inhaled carbon monoxide accelerates resolution of inflammation via unique proresolving mediator-heme oxygenase-1 circuits. J. Immunol..

[149] Seta F., Bellner L., Rezzani R., Regan R.F., Dunn M.W., Abraham N.G., Gronert K., Laniado-Schwartzman M. (2006). Heme oxygenase-2 is a critical determinant for execution of an acute inflammatory and reparative response. Am. J. Pathol..

[150] Bellner L., Martinelli L., Halilovic A., Patil K., Puri N., Dunn M.W., Regan R.F., Schwartzman M.L. (2009). Heme oxygenase-2 deletion causes endothelial cell activation marked by oxidative stress, inflammation, and angiogenesis. J. Pharmacol. Exp. Ther..

[151] Chen R.J., Yuan H.H., Zhang T.Y., Wang Z.Z., Hu A.K., Wu L.L., Yang Z.P., Mao Y.J., Ji D.J., Zhu X.R. (2014). Heme oxygenase-2 suppress TNF-α and IL6 expression via TLR4/MyD88-dependent signaling pathway in mouse cerebral vascular endothelial cells. Mol. Neurobiol..

[152] Basuroy S., Bhattacharya S., Tcheranova D., Qu Y., Regan R.F., Leffler C.W., Parfenova H. (2006). HO-2 provides endogenous protection against oxidative stress and apoptosis caused by TNF-alpha in cerebral vascular endothelial cells. Am. J. Physiol. Cell Physiol..

[153] Marrazzo G., Bellner L., Halilovic A., Li Volti G., Drago F., Dunn M.W., Schwartzman M.L. (2011). The role of neutrophils in corneal wound healing in HO-2 null mice. PLoS ONE.

[154] Lundvig D.M., Scharstuhl A., Cremers N.A., Pennings S.W., te Paske J., van Rheden R., van Run-van Breda C., Regan R.F., Russel F.G., Carels C.E. (2014). Delayed cutaneous wound closure in HO-2 deficient mice despite normal HO-1 expression. J. Cell Mol. Med..

[155] Chen R., Wang Z., Chen Q., Zhang T., Zhu Y., Xian X., Han X. (2018). Heme oxygenase-2 suppresses acute inflammation and improves the survival of skin allografts. Int. Immunopharmacol..

[156] Choi A.M., Ryter S.W., Levine B. (2013). Autophagy in human health and disease. N. Engl. J. Med..

[157] Mizushima N. (2018). A brief history of autophagy from cell biology to physiology and disease. Nat. Cell Biol..

[158] Mizushima N., Komatsu M. (2011). Autophagy: Renovation of cells and tissues. Cell.

[159] Rubinsztein D.C., Codogno P., Levine B. (2012). Autophagy modulation as a potential therapeutic target for diverse diseases. Nat. Rev. Drug Discov..

[160] Levine B., Kroemer G. (2019). Biological functions of autophagy genes: A disease perspective. Cell.

[161] Yin Z., Pascual C., Klionsky D.J. (2016). Autophagy: Machinery and regulation. Microb. Cell.

[162] Doherty J., Baehrecke E.H. (2018). Life, death and autophagy. Nat. Cell Biol..

[163] Miller D.R., Cramer S.D., Thorburn A. (2020). The interplay of autophagy and non-apoptotic cell death pathways. Int. Rev. Cell Mol. Biol..

[164] Stolz A., Ernst A., Dikic I. (2014). Cargo recognition and trafficking in selective autophagy. Nat. Cell Biol..

[165] Lamark T., Svenning S., Johansen T. (2017). Regulation of selective autophagy: The p62/SQSTM1 paradigm. Essays Biochem..

[166] Lippai M., Lőw P. (2014). The role of the selective adaptor p62 and ubiquitin-like proteins in autophagy. Biomed. Res. Int..

[167] Youle R.J., Narendra D.P. (2011). Mechanisms of mitophagy. Nat. Rev. Mol. Cell Biol..

[168] Lazarou M., Sliter D.A., Kane L.A., Sarraf S.A., Wang C., Burman J.L., Sideris D.P., Fogel A.I., Youle R.J. (2015). The ubiquitin kinase PINK1 recruits autophagy receptors to induce mitophagy. Nature..

[169] Lee I.H., Cao L., Mostoslavsky R., Lombard D.B., Liu J., Bruns N.E., Tsokos M., Alt F.W., Finkel T. (2008). A role for the NAD-dependent deacetylase Sirt1 in the regulation of autophagy. Proc. Natl. Acad. Sci. USA.

[170] Suliman H.B., Keenan J.E., Piantadosi C.A. (2017). Mitochondrial quality-control dysregulation in conditional HO-1(-/-) mice. JCI Insight..

[171] Kim H.P., Wang X., Chen Z.H., Lee S.J., Huang M.H., Wang Y., Ryter S.W., Choi A.M. (2008). Autophagic proteins regulate cigarette smoke-induced apoptosis: Protective role of heme oxygenase-1. Autophagy.

[172] Carchman E.H., Rao J., Loughran P.A., Rosengart M.R., Zuckerbraun B.S. (2011). Heme oxygenase-1-mediated autophagy protects against hepatocyte cell death and hepatic injury from infection/sepsis in mice. Hepatology.

[173] Peng Z., Liao Y., Wang X., Chen L., Wang L., Qin C., Wang Z., Cai M., Hu J., Li D. (2020). Heme oxygenase-1 regulates autophagy through carbon-oxygen to alleviate deoxynivalenol-induced hepatic damage. Arch. Toxicol..

[174] Liu A., Guo E., Yang J., Li R., Yang Y., Liu S., Hu J., Jiang X., Dirsch O., Dahmen U. (2016). Ischemic preconditioning attenuates ischemia/reperfusion injury in rat steatotic liver: Role of heme oxygenase-1-mediated autophagy. Oncotarget.

[175] Yun N., Cho H.I., Lee S.M. (2014). Impaired autophagy contributes to hepatocellular damage during ischemia/reperfusion: Heme oxygenase-1 as a possible regulator. Free Radic. Biol. Med..

[176] Nakamura K., Kageyama S., Yue S., Huang J., Fujii T., Ke B., Sosa R.A., Reed E.F., Datta N., Zarrinpar A. (2018). Heme oxygenase-1 regulates sirtuin-1-autophagy pathway in liver transplantation: From mouse to human. Am. J. Transplant..

[177] Waltz P., Carchman E.H., Young A.C., Rao J., Rosengart M.R., Kaczorowski D., Zuckerbraun B.S. (2011). Lipopolysaccaride induces autophagic signaling in macrophages via a TLR4, heme oxygenase-1 dependent pathway. Autophagy.

[178] Schwartz M., Böckmann S., Borchert P., Hinz B. (2018). SB202190 inhibits endothelial cell apoptosis via induction of autophagy and heme oxygenase-1. Oncotarget.

[179] Lee S.J., Ryter S.W., Xu J.F., Nakahira K., Kim H.P., Choi A.M., Kim Y.S. (2011). Carbon monoxide activates autophagy via mitochondrial reactive oxygen species formation. Am. J. Respir. Cell Mol. Biol..

[180] Lee S., Lee S.J., Coronata A.A., Fredenburgh L.E., Chung S.W., Perrella M.A., Nakahira K., Ryter S.W., Choi A.M. (2014). Carbon monoxide confers protection in sepsis by enhancing beclin 1-dependent autophagy and phagocytosis. Antioxid. Redox Signal..

[181] Chen S., Ding R., Hu Z., Yin X., Xiao F., Zhang W., Yan S., Lv C. (2020). MicroRNA-34a inhibition alleviates lung injury in cecal ligation and puncture Induced septic mice. Front. Immunol..

[182] Petrache I., Otterbein L.E., Alam J., Wiegand G.W., Choi A.M. (2000). Heme oxygenase-1 inhibits TNF-alpha-induced apoptosis in cultured fibroblasts. Am. J. Physiol. Lung Cell Mol. Physiol..

[183] Brouard S., Berberat P.O., Tobiasch E., Seldon M.P., Bach F.H., Soares M.P. (2002). Heme oxygenase-1-derived carbon monoxide requires the activation of transcription factor NF-kappa B to protect endothelial cells from tumor necrosis factor-alpha-mediated apoptosis. J. Biol. Chem..

[184] Liu X.M., Chapman G.B., Peyton K.J., Schafer A.I., Durante W. (2003). Antiapoptotic action of carbon monoxide on cultured vascular smooth muscle cells. Exp. Biol. Med..

[185] Liu X.M., Chapman G.B., Peyton K.J., Schafer A.I., Durante W. (2002). Carbon monoxide inhibits apoptosis in vascular smooth muscle cells. Cardiovasc. Res..

[186] Zhang X., Shan P., Otterbein L.E., Alam J., Flavell R.A., Davis R.J., Choi A.M., Lee P.J. (2003). Carbon monoxide inhibition of apoptosis during ischemia-reperfusion lung injury is dependent on the p38 mitogen-activated protein kinase pathway and involves caspase 3. J. Biol. Chem..

[187] Lamkanfi M., Dixit V.M. (2014). Mechanisms and functions of inflammasomes. Cell.

[188] Jorgensen I., Miao E.A. (2015). Pyroptotic cell death defends against intracellular pathogens. Immunol. Rev..

[189] Li H.B., Zhang X.Z., Sun Y., Zhou Q., Song J.N., Hu Z.F., Li Y., Wu J.N., Guo Y., Zhang Y. (2020). HO-1/PINK1 Regulated mitochondrial fusion/fission to inhibit pyroptosis and attenuate septic acute kidney injury. Biomed. Res. Int..

[190] Diao C., Chen Z., Qiu T., Liu H., Yang Y., Liu X., Wu J., Wang L. (2019). Inhibition of PRMT5 attenuates oxidative stress-induced pyroptosis via activation of the Nrf2/HO-1 signal pathway in a mouse model of Renal Ischemia-Reperfusion Injury. Oxid. Med. Cell Longev..

[191] Fei L., Jingyuan X., Fangte L., Huijun D., Liu Y., Ren J., Jinyuan L., Linghui P. (2020). Preconditioning with rHMGB1 ameliorates lung ischemia-reperfusion injury by inhibiting alveolar macrophage pyroptosis via the Keap1/Nrf2/HO-1 signaling pathway. J. Transl. Med..

[192] Fu L., Zhang D.X., Zhang L.M., Song Y.C., Liu F.H., Li Y., Wang X.P., Zheng W.C., Wang X.D., Gui C.X. (2020). Exogenous carbon monoxide protects against mitochondrial DNA-induced hippocampal pyroptosis in a model of hemorrhagic shock and resuscitation. Int. J. Mol. Med..

[193] Galluzzi L., Kepp O., Chan F.K., Kroemer G. (2017). Necroptosis: Mechanisms and relevance to disease. Annu. Rev. Pathol..

[194] Choi M.E., Price D.R., Ryter S.W., Choi A.M.K. (2019). Necroptosis: A crucial pathogenic mediator of human disease. JCI Insight..

[195] Linkermann A., Green D.R. (2014). Necroptosis. N. Engl. J. Med..

[196] Kaczmarek A., Vandenabeele P., Krysko D.V. (2013). Necroptosis: The release of damage-associated molecular patterns and its physiological relevance. Immunity..

[197] Tonnus W., Meyer C., Paliege A., Belavgeni A., von Mässenhausen A., Bornstein S.R., Hugo C., Becker J.U., Linkermann A. (2019). The pathological features of regulated necrosis. J. Pathol..

[198] Orozco S., Oberst A. (2017). RIPK3 in cell death and inflammation: The good, the bad, and the ugly. Immunol. Rev..

[199] Pasparakis M., Vandenabeele P. (2015). Necroptosis and its role in inflammation. Nature.

[200] Weinlich R., Oberst A., Beere H.M., Green D.R. (2017). Necroptosis in development, inflammation and disease. Nat. Rev. Mol. Cell Biol..

[201] Chen J., Kos R., Garssen J., Redegeld F. (2019). Molecular insights into the mechanism of necroptosis: The necrosome as a potential therapeutic target. Cells.

[202] Fortes G.B., Alves L.S., de Oliveira R., Dutra F.F., Rodrigues D., Fernandez P.L., Souto-Padron T., De Rosa M.J., Kelliher M., Golenbock D. (2012). Heme induces programmed necrosis on macrophages through autocrine TNF and ROS production. Blood.

[203] Chenxu G., Minxuan X., Yuting Q., Tingting G., Jing F., Jinxiao L., Sujun W., Yongjie M., Deshuai L., Qiang L. (2019). Loss of RIP3 initiates annihilation of high-fat diet initialized nonalcoholic hepatosteatosis: A mechanism involving Toll-like receptor 4 and oxidative stress. Free Radic. Biol. Med..

[204] Germanova D., Keirsse J., Köhler A., Hastir J.F., Demetter P., Delbauve S., Elkrim Y., Verset L., Larbanoix L., Preyat N. (2021). Myeloid tumor necrosis factor and heme oxygenase-1 regulate the progression of colorectal liver metastases during hepatic ischemia-reperfusion. Int. J. Cancer.

[205] Yin W., Wang C., Peng Y., Yuan W., Zhang Z., Liu H., Xia Z., Ren C., Qian J. (2020). Dexmedetomidine alleviates H^2^O^2^-induced oxidative stress and cell necroptosis through activating of α2-adrenoceptor in H9C2 cells. Mol. Biol. Rep..

[206] Dixon S.J., Lemberg K.M., Lamprecht M.R., Skouta R., Zaitsev E.M., Gleason C.E., Patel D.N., Bauer A.J., Cantley A.M., Yang W.S. (2012). Ferroptosis: An iron-dependent form of nonapoptotic cell death. Cell.

[207] Sun Y., Chen P., Zhai B., Zhang M., Xiang Y., Fang J., Xu S., Gao Y., Chen X., Sui X. (2020). The emerging role of ferroptosis in inflammation. Biomed. Pharmacother..

[208] Imai H., Matsuoka M., Kumagai T., Sakamoto T., Koumura T. (2017). Lipid peroxidation-dependent cell death regulated by GPx4 and ferroptosis. Curr. Top. Microbiol. Immunol..

[209] Latunde-Dada G.O. (2017). Ferroptosis: Role of lipid peroxidation, iron and ferritinophagy. Biochim. Biophys. Acta Gen. Subj..

[210] Hou W., Xie Y., Song X., Sun X., Lotze M.T., Zeh H.J., Kang R., Tang D. (2016). Autophagy promotes ferroptosis by degradation of ferritin. Autophagy.

[211] Kajarabille N., Latunde-Dada G.O. (2019). Programmed cell-death by ferroptosis: Antioxidants as mitigators. Int. J. Mol. Sci..

[212] Yu H., Guo P., Xie X., Wang Y., Chen G. (2017). Ferroptosis, a new form of cell death, and its relationships with tumourous diseases. J. Cell. Mol. Med..

[213] Ryter S.W., Si M., Lai C.C., Su C.Y. (2000). Regulation of endothelial heme oxygenase activity during hypoxia is dependent on chelatable iron. Am. J. Physiol. Heart Circ. Physiol..

[214] Keyse S.M., Tyrrell R.M. (1990). Induction of the heme oxygenase gene in human skin fibroblasts by hydrogen peroxide and UVA (365 nm) radiation: Evidence for the involvement of the hydroxyl radical. Carcinogenesis.

[215] Fang X., Wang H., Han D., Xie E., Yang X., Wei J., Gu S., Gao F., Zhu N., Yin X. (2019). Ferroptosis as a target for protection against cardiomyopathy. Proc. Natl. Acad. Sci. USA.

[216] Bao W.D., Zhou X.T., Zhou L.T., Wang F., Yin X., Lu Y., Zhu L.Q., Liu D. (2020). Targeting miR-124/Ferroportin signaling ameliorated neuronal cell death through inhibiting apoptosis and ferroptosis in aged intracerebral hemorrhage murine model. Aging Cell.

[217] Kwon M.Y., Park E., Lee S.J., Chung S.W. (2015). Heme oxygenase-1 accelerates erastin-induced ferroptotic cell death. Oncotarget.

[218] Chang L.C., Chiang S.K., Chen S.E., Yu Y.L., Chou R.H., Chang W.C. (2018). Heme oxygenase-1 mediates BAY 11-7085 induced ferroptosis. Cancer Lett..

[219] Guerrero-Hue M., García-Caballero C., Palomino-Antolín A., Rubio-Navarro A., Vázquez-Carballo C., Herencia C., Martín-Sanchez D., Farré-Alins V., Egea J., Cannata P. (2019). Curcumin reduces renal damage associated with rhabdomyolysis by decreasing ferroptosis-mediated cell death. FASEB J..

[220] Adedoyin O., Boddu R., Traylor A., Lever J.M., Bolisetty S., George J.F., Agarwal A. (2018). Heme oxygenase-1 mitigates ferroptosis in renal proximal tubule cells. Am. J. Physiol. Renal Physiol..

[221] Ryter S.W. (2020). Therapeutic potential of heme oxygenase-1 and carbon monoxide in acute organ injury, critical illness, and inflammatory disorders. Antioxidants.

[222] Vasconcellos L.R., Siqueira M.S., Moraes R., Carneiro L.A., Bozza M.T., Travassos L.H. (2018). Heme oxygenase-1 and autophagy linked for cytoprotection. Curr. Pharm. Des..

[223] Singh S.P., Greenberg M., Glick Y., Bellner L., Favero G., Rezzani R., Rodella L.F., Agostinucci K., Shapiro J.I., Abraham N.G. (2020). Adipocyte specific HO-1 gene therapy is effective in antioxidant treatment of insulin resistance and vascular function in an obese mice model. Antioxidants.

[224] Abraham N.G., Asija A., Drummond G., Peterson S. (2007). Heme oxygenase-1 gene therapy: Recent advances and therapeutic applications. Curr. Gene Ther..

[225] Otterbein L.E., Soares M.P., Yamashita K., Bach F.H. (2003). Heme oxygenase-1: Unleashing the protective properties of heme. Trends Immunol..

[226] Ling K., Men F., Wang W.C., Zhou Y.Q., Zhang H.W., Ye D.W. (2018). Carbon monoxide and its controlled release: Therapeutic application, detection, and development of carbon monoxide releasing molecules (CORMs). J. Med. Chem..

[227] Steiger C., Hermann C., Meinel L. (2017). Localized delivery of carbon monoxide. Eur. J. Pharm. Biopharm..

[228] Lazarus L.S., Benninghoff A.D., Berreau L.M. (2020). Development of triggerable, trackable, and targetable carbon monoxide releasing molecules. Acc. Chem. Res..

[229] Yang X.X., Ke B.W., Lu W., Wang B.H. (2020). CO as a therapeutic agent: Discovery and delivery forms. Chin. J. Nat. Med..

